# Overlapping Structures in Sensory-Motor Mappings

**DOI:** 10.1371/journal.pone.0084240

**Published:** 2014-01-02

**Authors:** Kevin Earland, Mark Lee, Patricia Shaw, James Law

**Affiliations:** Department of Computer Science/Aberystwith University, Wales, United Kingdom; The University of Plymouth, United Kingdom

## Abstract

This paper examines a biologically-inspired representation technique designed for the support of sensory-motor learning in developmental robotics. An interesting feature of the many topographic neural sheets in the brain is that closely packed receptive fields must overlap in order to fully cover a spatial region. This raises interesting scientific questions with engineering implications: e.g. is overlap detrimental? does it have any benefits? This paper examines the effects and properties of overlap between elements arranged in arrays or maps. In particular we investigate how overlap affects the representation and transmission of spatial location information on and between topographic maps. Through a series of experiments we determine the conditions under which overlap offers advantages and identify useful ranges of overlap for building mappings in cognitive robotic systems. Our motivation is to understand the phenomena of overlap in order to provide guidance for application in sensory-motor learning robots.

## Introduction

Many of the sensors in the human body and the neurons in the central nervous system effectively capture their inputs over a spatial region rather than at a specific point. These *receptive fields* have smooth curved convex boundaries and so approximate to distorted circular shapes. A notable feature of such shapes when packed together, as in topographic sheets, is that they must overlap in order to fully cover a spatial area — unlike the arrays of contiguous pixels used in digital systems. Thus, overlap implies at least partial sharing of inputs in arrays of fields. This phenomenon of overlap raises interesting scientific questions with engineering implications: are there any benefits with overlapping structures? what function could overlap serve? how is accuracy affected by overlap in an array? what is the best size of the fields for a given task? It might appear at first that overlap introduces unnecessary crosstalk and reduces accuracy. However, we have used overlapping fields very successfully in a series of sensory-motor mapping experiments on real robots and this paper examines some of their properties and effects. The question we address here concerns how overlap affects the representation and transmission of spatial location information on and between topographic maps. The aim is to better understand overlap, particularly in the context of spatial localisation and cross-modal coordination, in order to build more efficient representation models for application in sensory-motor robot learning systems.

Actual or effective overlap occurs in many neuronal and sensing mechanisms in biology. For example, sensory receptors often project divergently onto higher-order layers of neurons and motor signals usually converge through neural layers, in both cases single cells receive inputs from increasing numbers of neighbouring regions [1], [2]. The sensors need not physically overlap; for example, in the eye the rods and cones connect to bipolar, horizontal and amacrine cells which then connect to the ganglion cells that exit the eye and form the optic nerve to the brain [3]. As well as providing various important visual functions, the overlapping fields of the interneuron cells create effective structural overlap between the sensors and the ganglion output. Structural overlap is not the only way that overlapping effects can occur, for example, the eye is subject to a constant high frequency (80 Hz) vibration (the ocular microtremor) that has the effect of causing stimuli points to overlap [4]. This is functionally important because when the microtremor is artificially suppressed, by image stabilisation, visual perception fades and disappears. A few authors have pointed out that edge-detection quality can be improved when receptive fields overlap as compared with conventional contiguous image cells [4], [5]. Considerable theoretical work has been done on possible models for the growth and plasticity of topological maps in the brain, e.g. [6], and these explore the tradeoffs between factors such as coverage and continuity.

It is clear that computational maps exist in the brain that can produce highly efficient forms of information processing [7]. But while there is much inspiration to be gained from these studies, there is little guidance to help robot implementors and experimenters to design adequate and efficient models for particular representations and cognitive tasks. This is especially true for the phenomenon of overlapping fields and we explore the following questions; what are the effects of overlap on locational accuracy in a topographic array and on mappings between two topographic arrays8 how does overlap influence the structure of connections between arrays? and, do overlapping fields have a useful role in representing transformations between arrays? Our focus throughout is on spatial information, that is, locative information about a stimulus point in a sensory or motor space. We have examined these issues by building an abstract, simplified model with significant overlapping elements and then investigated performance through an intensive programme of simulation experiments. Although it is possible to explore regular structures by analytical means we wished to include unstructured arrays and random elements and compare these with the regular cases. For this reason we used simulation as a general tool throughout, and all the results are taken from the mean performance over many simulation trials.

This paper is organised as follows. In the next section we define the general model and mathematical nature of our topographical mapping method for the representation of sensory-motor transforms and coordination structures. Then we describe a series of experiments on an implementation of the model in order to explore the effects of overlap in terms of accuracy, noise and transform fidelity. Next we present a series of the most significant results from those experiments along with results from two example robotic applications. The paper ends with a discussion of the findings and a brief comparison of the conclusions with those drawn from studies of overlap in living neural systems.

## A Sensory-Motor Mapping Model

It is important to state the assumptions and simplifications used in our analysis. The models used here are intended to be sufficiently abstract to allow reasonable generality in focusing on the role of overlap between modules while reducing complications from other sources. We focus on the transfer of spatial information between layers of neuron-like modules but do not stipulate any detailed internal structure of those modules. We assume independence between the modules, i.e. any inter-field computational effects are ignored. Our interest is in the spatial effects of overlap, rather than the processing of stimuli features, and stimuli are modelled as point excitations at discrete spatial locations. Although we do not attempt to model any biological systems perhaps the best inspiration comes from the mapping between the retina and the Superior Colliculus where notable correspondence is evident but is distorted in a non-linear manner [8]. We reason that a desired micro structure of the modules, or any enhancing superstructure, can be superimposed on our simplified abstraction without seriously affecting the underlying conclusions about overlapping effects. For example, the centre-surround receptive field structures found in visual and somatosensory systems provide both increased sensitivity and improved spatial localisation, but we ignore this subfield complexity in our simple modules as we wish to separate the details of such complex responses from the phenomena of local overlap with neighbours. Hence we define our modules as independent processors of their inputs and allow their resultant responses to overlap.

### Maps and mappings

In several previously reported experiments [9]–[11], we have used two-dimensional arrays of overlapping elements with explicit links between corresponding sensory or motor values for the representation of sensory-motor transforms and coordination structures.

Although three dimensions might seem appropriate for representing spatial events, we take inspiration from neuroscience, which shows that most areas of the brain are organised in topographical two-dimensional layers [12], [13]. This remarkable structural consistency suggests some potential advantage or efficacy in such two-dimensional arrangements [14]. We base our experiments on this scenario but, as demonstrated later, the techniques described also work for higher dimensional spaces.

A typical coordination structure will consist of a 2D array representing two sensory or motor variables, known as a *map* or *surface*, connected to another 2D array by a set of *links* that join points or small regions, known as *fields* by analogy with receptive fields, in each array.

More formally, let there exist a sensory system 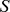 with two independent variables, 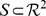, and a motor system 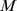 also describable in terms of two variables, 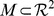. Then a *mapping* 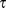 can be defined as:
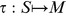
(1)


where the set members in S are either points, 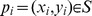, or local regions of the surface, 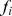, i.e. *fields*. Each field has a reference point or *centre*, 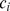, and a *boundary* defined by a boundary function 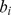, so that the field 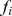 consists of all the points 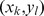 inside the boundary 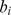. The surface 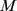 is similarly covered with points or fields.

We will use the above sensory-motor example throughout but all that follows equally applies to any intermediate maps, with possibly very indirect connections to sensory or motor systems. We will often refer to input values as *stimuli*. Finally, note that it is possible for mappings to be bidirectional (i.e. given a value from 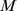 we can find an associated value from 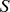), unlike most neural network models.

### Mappings and their growth

Spatial coordination is a significant issue in both neuroscience and robotics research because it is necessary to coordinate the differing spatial frameworks of the various sensory and motor systems. For example, coordinating the spatial frame of an active vision system with the spatial structure of a hand/arm system requires cross-modal relations to be established and understood; in this case, image based information needs to be related to the coordinate data available from a multi-degree of freedom mechanism.

**Figure 1:**
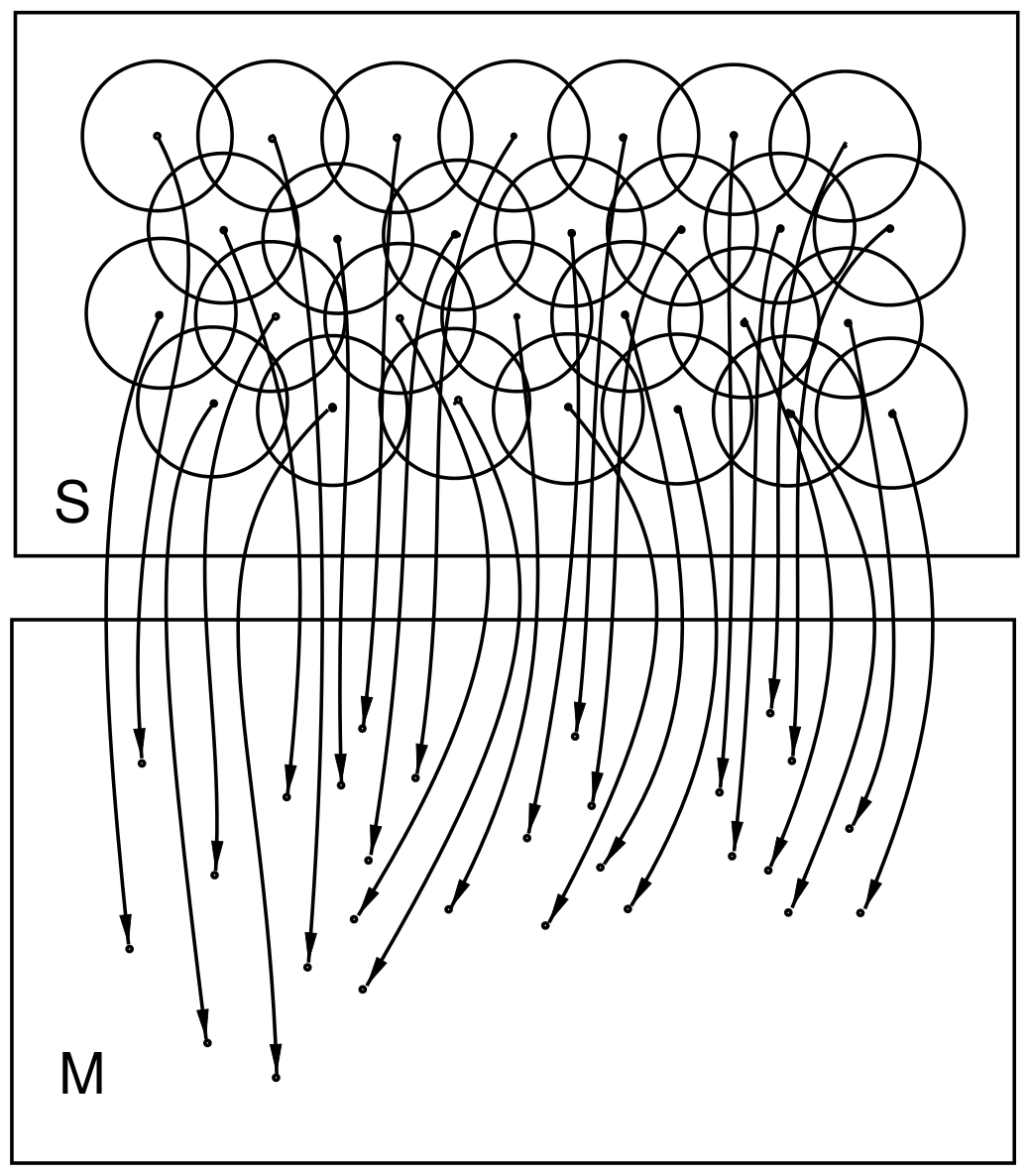
Example mapping. An 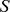 to 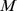 mapping with many-to-one structure.

As a simple example consider a saccade system for an eyeball (or active camera). A stimulus on the periphery of the retina (image) is to be brought to the fovea (centre) by a change in the eyeball (camera) gaze orientation. This requires a relation between the 2D image on the retina and the 2D motor system consisting of the two degrees of freedom provided by the two axes of movement of the eyeball (or camera). One solution could involve two surfaces, 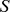 and 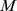, representing the retina and the motor components respectively, and explicit links from peripheral fields in 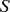 could access the appropriate motor values in 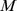 that will drive the eye such that the peripheral stimulus point becomes the centre of gaze. For a complete mapping we would expect every point in 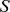 to be covered by at least one field that links to a suitable motor vector given by a point in 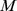. Figure 1 shows an illustration of a complete mapping for 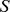. The radii are low for clarity and this gives very little overlap. Note that this case is a many-to-one mapping, as 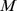 contains points not fields, and values between the points can be determined if necessary by an interpolation method. Also note that not all of 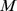 needs to be covered; mappings will often cover a different space on 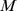 than on 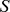.

**Figure 2:**
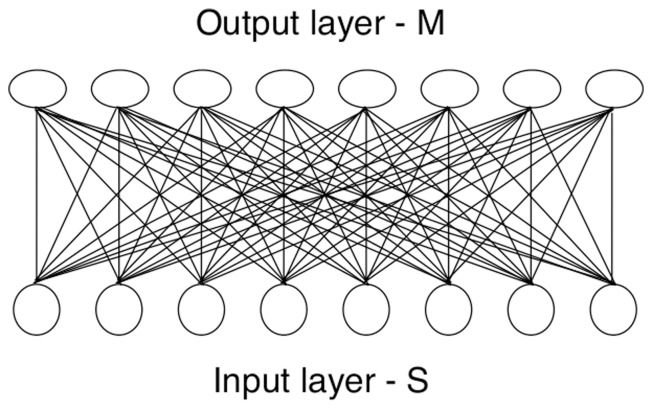
Total connectivity. Typical structure in artificial neural nets.

There are several possibilities for mechanisms that could establish the links between 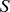 and 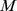. Conventional connectionist practice might advocate the provision of weighted links from each field on 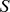 to every field on 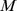, as in figure 2, with the path strength for each link being stored in a weight. As stimuli are experienced so the weights are adjusted to reflect the usage value of each link in representing the emerging mapping. After the weights have been adjusted many times the distribution of the weights then records the correlation pattern. There is some superficial justification for such a scheme because early neurogenesis massively overproduces synaptic connections [1] which are then pruned down during early neonatal experience [15]. However, the idea that there could be complete connectivety between maps has been shown to be totally unrealistic for the brain [16]. Also this method would be very inefficient for sensory-motor coordination because for any reasonable mapping the vast majority of the weights would be zero, giving a proportion of unused links much higher than even the 50% pruning rates reported for early cortical development [17]. Furthermore, before the maps can be used, this approach requires a (long) learning period while the weights converge; this is also infeasible.

We favour an alternative method in which the links are created at the point when an association between two maps is experienced. Thus, when a positive spatiotemporal correlation occurs between a pair of events (e.g. the target location on the periphery of the retina and the motor vector that is appropriate to bring the eye to fixate on that target) an explicit link is established between the respective points or fields on the 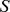 and 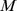 maps. This has the advantage that the mapping grows with experience and becomes structured to match the pattern of the correlations. It is also real-time, cumulative and incremental; all being important features for robotic and developmental models.

### Fields and their structure

It is important that our terminology is not confused with *field computation* in which large numbers of computational elements can be considered as continuous distributions of data. Mathematical techniques for continuous fields have found wide application in the physical sciences, e.g. for the analysis of flow of heat, fluids and stress forces, and they are now being used in brain modelling and neuroscience [18]. These studies are different in that they mainly focus on large scale effects, rather than local overlap, but we note that continuous techniques can be used to express linear projections between maps [18].

#### Field sizes and shapes

The concept of a field is meant to capture the idea of local spatial equivalence or influence surrounding discrete neural modules. In a two-dimensional sensory system, 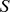, a stimulus might be defined by its point of occurrence, 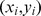, but the accuracy and resolution of both biological and artificial sensing (and motor) systems are finite and in practice all points within a local region, e.g. 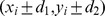, will be indistinguishable. Here the 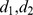 can be seen as *tolerance* parameters that define a locality within which stimuli are deemed equivalent. These two parameters give a simple boundary function but such rectangular shaped fields are awkward and not neurologically valid [13]. A better field model is an elliptical or circular boundary function; thus by defining a radial distance 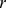 from the field centre 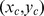, a stimulus at 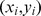 can be detected by the field if 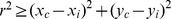.

#### Field distributions and overlap

Fields can be distributed across maps either in a structured or an unstructured manner. We can examine these options by considering the field centres either to be aligned with a regular lattice or to be randomly placed. First, considering the structured case, we note that to build a regular lattice there are only three possible shapes that can tesselate the plane: square, triangle, and hexagon. We do not consider the hexagonal case as this produces a lattice which is a subset of the triangular case.

**Figure 3:**
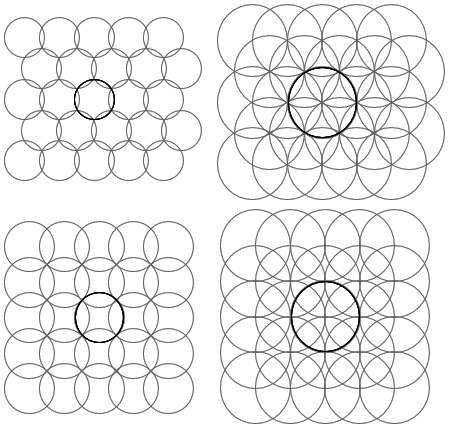
Structure comparison. Comparison of arrays of fields centred on triangular (top row) and square (bottom row) 5 by 5 grids. The left column shows minimum radii for complete coverage at 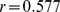 for triangular (top left) and 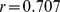 for square (bottom left). The right column shows 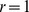. The central fields are highlighted for clarity of overlap.

If the plane is to be covered with circles then locating their centres on a triangular grid gives a more efficient covering (i.e. less wasted space without overlap) than using a square grid. Hence, for a uniform distribution of field centres in two axes, the fields should be placed on an equilateral triangular grid as, for example, the triangular structures in figure 3 (top row).

This can be compared with a rectangular grid, as normally used for image pixels. The minimum radius to ensure complete covering on a square grid of unit spacing is 0.707 and this gives 57% overlap (i.e. for any field only 43% of its area is not shared with another field); while the minimum radius for complete coverage on a triangular grid of unit spacing is 0.577, giving an area of overlap of only 21%, this is illustrated in figure 3 (left column).

**Figure 4:**
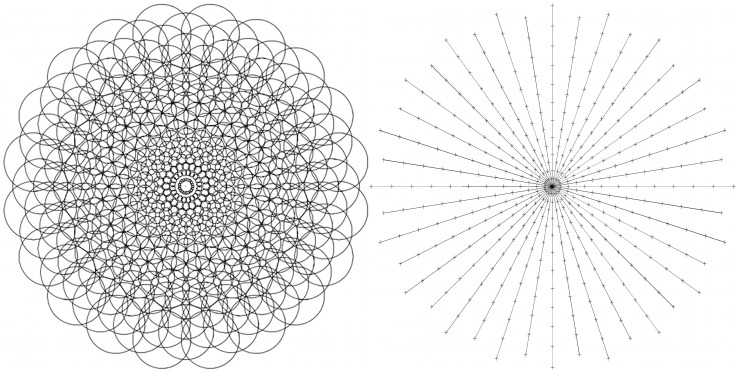
Polar structure. A polar field array (left) and a plot of the field centres on their radials (right).

Figure 4 (left) shows a field covering designed for an artificial retina. Here the peripheral fields increase in size in proportion to distance from the fovea (centre point). Notice from figure 4 (right) that the tessellation is nearer triangular locally rather than rectangular by arranging the fields on every other radial to be offset. This example shows how a field distribution can be arranged for a particular sensory structure with known requirements. In general, there are many options for determining field placements, using different formula or structures. See [19] for the design of overlapping field arrays for modelling the human retina and discussion of the lack of any exact analytic or geometric models.

**Figure 5:**
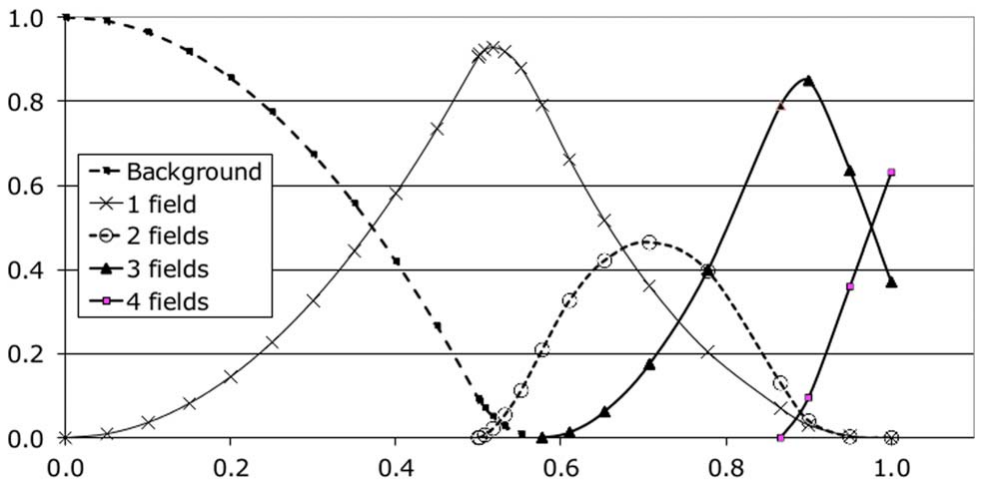
Field coverage. Areas of overlap with increasing field radius. Abscissa is field radius; ordinate gives coverage per unit area of the 2D plane.

**Figure 6:**
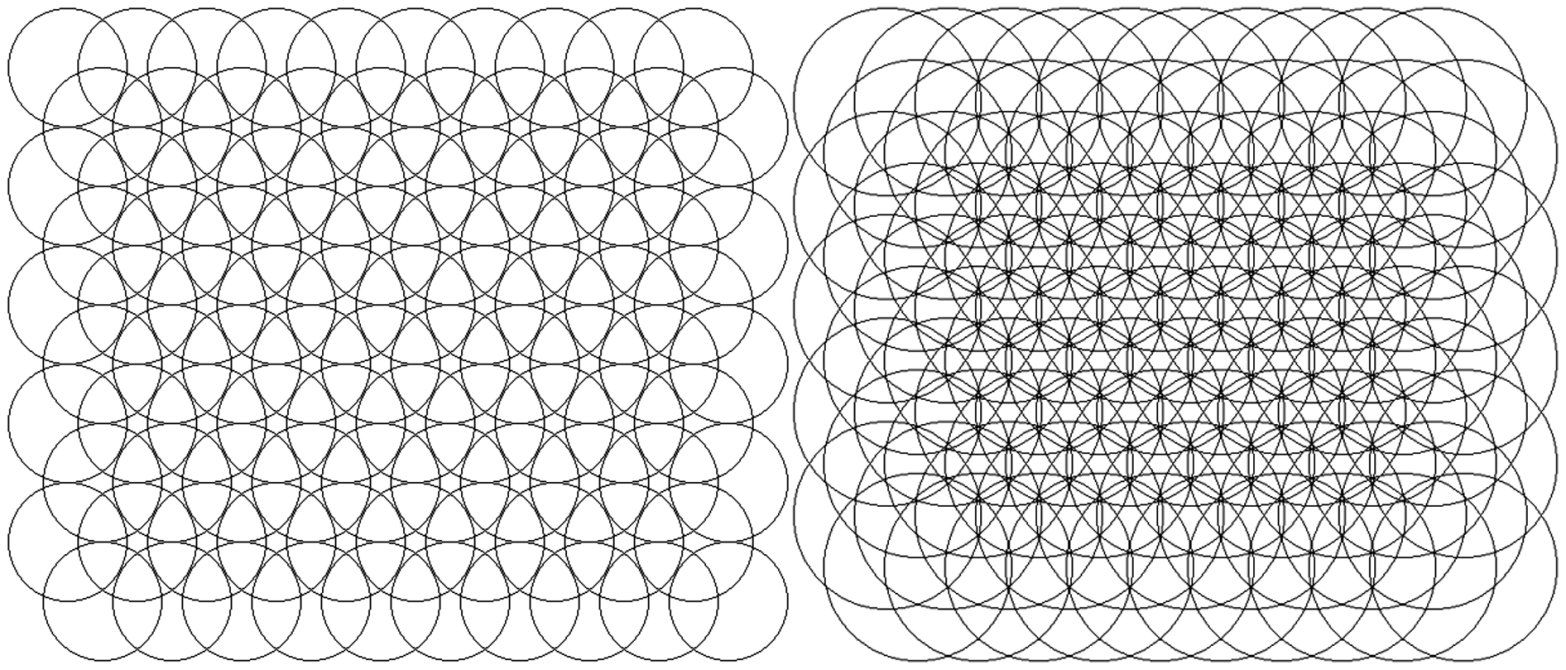
Overlap examples. Field overlap on regular triangular mesh, 10×10 fields, 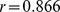 (left) and 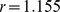 (right)

It is useful to understand how the density of overlap increases as fields are packed closer together. Figure 5 shows overlap in the 2D plane varying with increasing field size. All the data-points for figure 5 were obtained by exact calculation from geometric analysis. A uniform triangular grid of unit size is used, (as in figure 3, left column), and each grid point is the centre of a field of radius 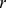. As 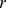 increases so the areas of overlap intensify. The leftmost peak is the background (i.e. the area not covered by any fields) and this decreases from 100% with no fields (at 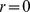) while the plot for the area covered by one field correspondingly increases. At 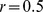 the point is reached where pairs of field boundaries are touching and where overlap between fields can begin. This is the optimum packing configuration for filling the plane with circles without overlap. As 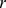 increases further, the area covered by overlap between two fields increases, while the area covered by single fields starts to decline. When 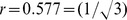 triples of field boundaries are now touching and the background reaches 0% (all points on the surface are covered). This is the start of three field overlap and any further increase in 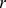 will see increasing area covered by three fields. Eventually the area of two-field overlaps reaches its peak and then declines towards zero, while the plot for three-field overlap builds towards another peak. At 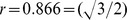 nearly all of the surface is covered by three-field overlaps and four field overlaps are just about to begin, (see figure 6, left). The state for 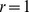 is notable as only 3 and 4 field overlaps exist; single and double coverage has finished and the next higher overlaps are just about to start. This pattern continues and the rate of growth of overlapping complexity is quite rapid with increasing 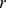; for example at 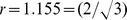 there are 4, 5, 6 and 7 fold overlaps, (shown in figure 6, right).

Pre-structured grids may seem inappropriate for developing systems but we note that regular grids do not preclude highly distorted mappings as the fields are *selected from* the grid and the full lattice structure is not necessarily imposed on the eventual mapping. There exists evidence that the topographic structure is determined during neurogenesis by many influences and *both* genetic and experiential inputs have strong effects [20]. It is possible that genetically encoded neural growth patterns provide regular arrays of neural sheets and then the interconnections are established by a separate process of coordination. There is also evidence that neurons can expand their receptive fields in order to adapt to a damaged area caused by a lesion [21] and we note that such plasticity is better served by a uniform grid structure rather than an irregular covering of fields.

## Methods

All experiments are based on a software implementation of the model described in the previous section. In most cases the fields are uniform (all circular of radius 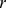) and are either structured, with field centres on an equilateral triangular grid, or randomly located to simulate unstructured generation.

In order to keep the results independent of the actual sizes used, the field radii are always reported as relative to the spacing so that 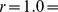 distance between field centres for the triangular grid structure.

In order to generate sets of unstructured field locations we ran many experiments to obtain very similar densities (fields per unit area) to a regular triangular layout and found this required the fields to be treated as if the radius was set to 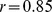. This permits reasonable comparison between the results for structured and unstructured placement.

### Field response functions

A receptive field usually has a central point of maximum response and the output can be defined in terms of the relation between the stimulus and this reference point. We define circular fields 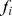 such that the output response varies according to the distance 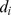 of the stimulus 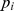 from the field centre. Then 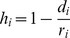
(2)is the offset distance, varying from 1.0 to zero as the stimulus moves from the centre to the field edge. The output can then be modulated by a function; 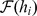. Several fields may be excited by a single stimulus and so these response functions produce an encoding of the spatial location of the stimulus. The nature of this encoding depends upon the response function and we consider several cases:

#### Uniform or flat response

The simplest case is to allow all stimulus points equal status and so all stimuli give the same effect as they would have at the centre point, thus: 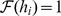. This gives a step function with sharp edges to the fields and ignores the location of the stimulus within the field.

#### Linear falloff

The response could be linearly reduced from 1.0 at the centre to zero at the field edge. Thus, the field signal is simply 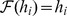. This gives a sharp, non-differentiable, peak at the centre of the field.

#### Nonlinear falloff

The biologically undesirable discontinuities of the above two cases can be removed with smooth, continuous functions, e.g. 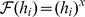. These can also give more rapid falloff. We experimented with many forms and selected three of the most interesting: a Gaussian response, 
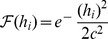
(3)


a cosine function, 
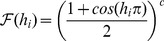
(4)


and a sigmoid function, 
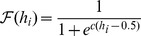
(5)


**Figure 7:**
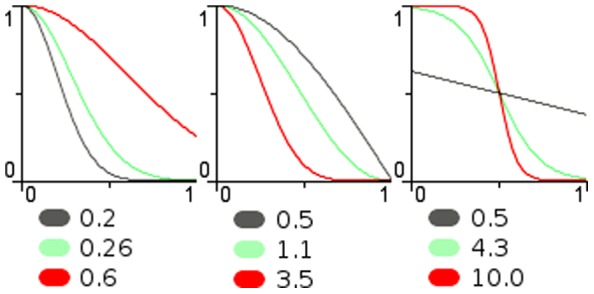
Nonlinear response functions. For each function, the input (normalised distance from the field centre) is along the abscissa and the output response is along the ordinate. Left shows a Gaussian function with coefficient 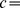 0.2, 0.26 and 0.6. Centre shows a cosine function with 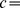 0.5, 1.1 and 3.5. Right shows a sigmoid function with 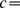 0.5, 4.3 and 10.0. All values are normalised to 1.

In each case the coefficient 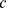 is used as the width control parameter. Figure 7 illustrates the behaviour of these functions.

Some of our experiments introduced noise into these functions so that the noise tolerance of the decoding technique can be characterised. If 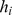 is a response value, 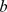 is a noise coefficient and 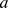 is a randomly generated value with Gaussian distribution scaled such that values with 3 times the standard deviation (approximately 99.7%) range from −1 to 1, then the noisy response value is: 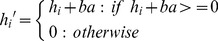
(6)


Another illustration of field activity is provided by allowing only one field to be active at a time. For this “single neuron” response we select the field with its centre nearest to the stimulus point 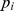.

### Field and link generation

**Figure 8:**
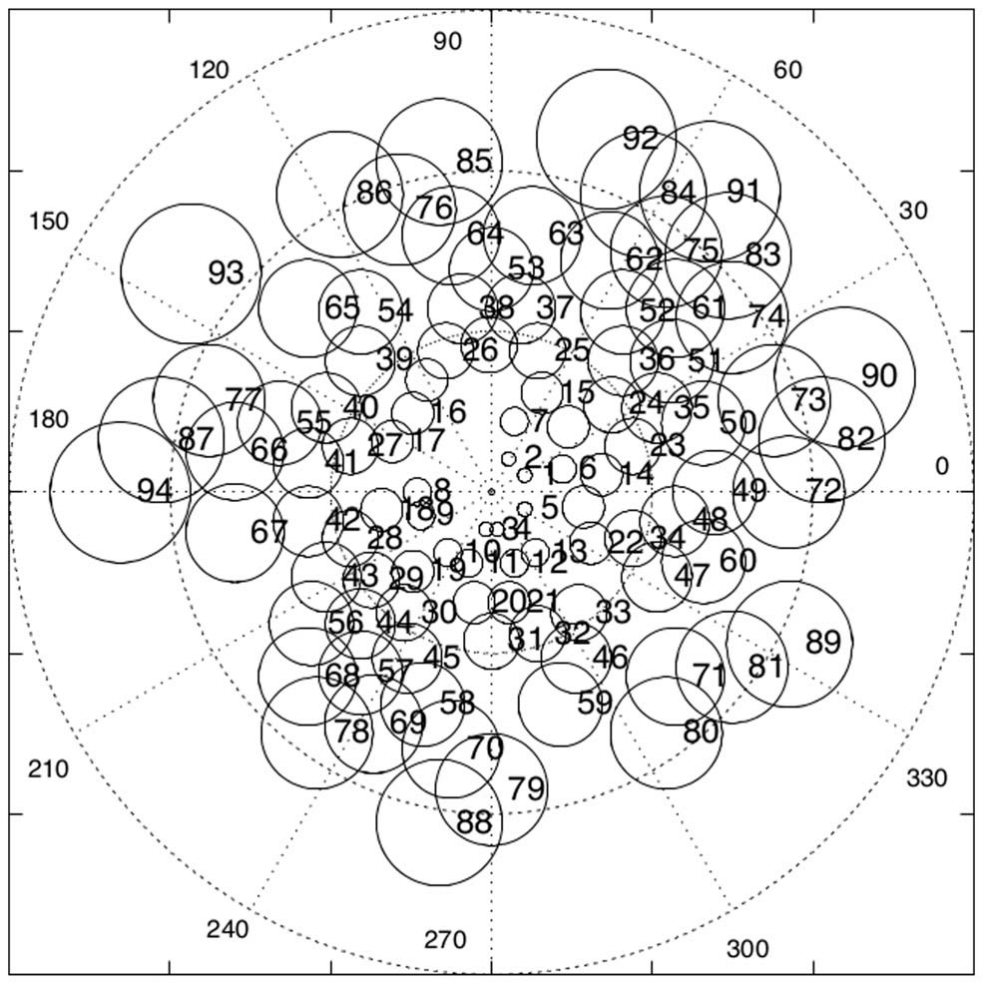
Partial population. Fields being generated from a structured polar grid.

Assume that a learning process generates stimulus points, 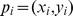, on a two-dimensional surface, 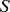, which is initially empty. If a stimulus point is already covered by a field on 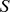, i.e. is within the radius of some existing field, then no action is required. But if 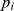 is not covered then a new field must be generated for this location. Fields may be structured or unstructured: either they can be selected from a prior pattern or they can be generated independently as they occur. In the former case, the field on the grid with the nearest centre to the uncovered point 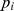 is selected and a link to 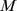 is created. In the latter case, a field is generated with its centre located at the exact stimulus point 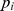. Eventually all points should be covered by one or more fields. If the grid of fields has low levels of overlap (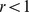), for example as in figure 3 (left), then there will always be places covered by only one field and so eventually all fields in the grid will be used. Conversely, with large overlap many of the possible fields will not need to be generated. Figure 4 showed a complex field grid for a retina design, with a great deal of overlap, but, in practice, only part of this may need to be generated. Figure 8 shows this during a learning experiment; fields are taken from the grid as needed and this covering process stops when every possible stimulus point has been covered.

### Decoding

**Figure 9:**
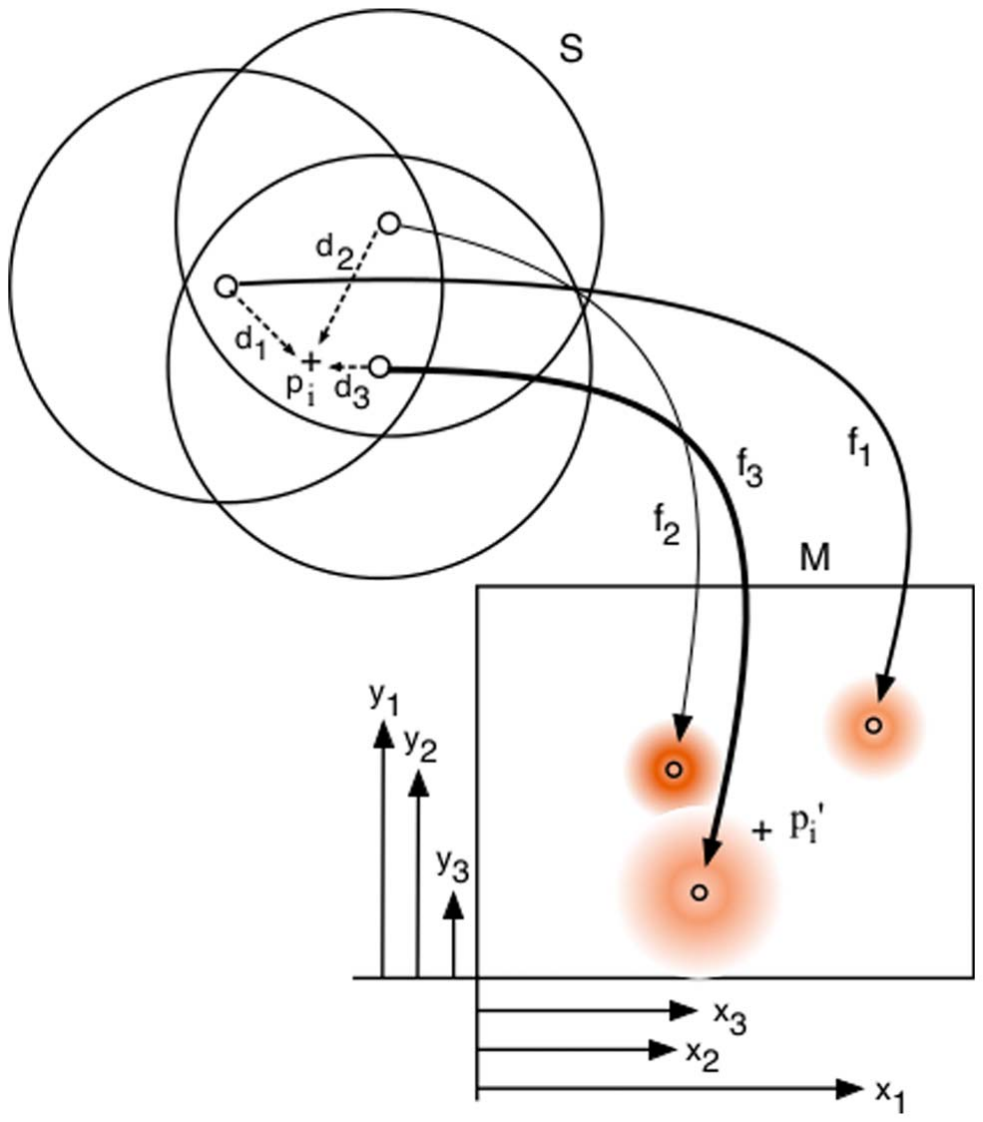
The decoding problem. Three fields cover the stimulus point and so three links are excited, each in accordance with their offset distance, 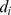. These values are transmitted to points on 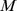, with the thickness of the lines and the halos around the points indicates the relative strengths of the signals. The values on 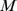 can then be combined by various possible mechanisms to identify a new resultant point 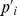.

When a stimulus point is covered by a field then that field is activated according to the proximity of the stimulus to the field centre. If a stimulus is covered by only one field then there will be only one link activated and the associated value on 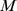 gives the result. However, if a stimulus point is in a region of overlap then several fields will be active and so several points on 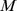 must be combined to give a single response value. This reverse process of finding a single result from a set of variably excited points or modules is known as decoding. Figure 9 illustrates decoding.

The main methods proposed for neural decoding are based on vector summation or vector averaging [22], [23]. These are inspired by large populations of active neurons [24], e.g. in the superior colliculus, which encode a set of directional signals [25]. We adopt this method whereby a set of points 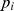 can be viewed as vectors, from a common reference, and then a weighted vector average is given by: 
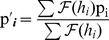
(7)


We also notice that trilateration or multilateration techniques [26] could be used for decoding. Trilateration is a method of locating an unknown point given its distances from three known points and is the dual of triangulation. Trilateration is attractive because theoretically it can provide an accurate evaluation on any location within the region defined by the three (or more) reference points. For trilateration to work properly it is important that the reference points form a triangle and not a line. When necessary we used Delaunay triangulation in our experiments to group the points into triangles and avoid thin lines [27].

### Measurements

We analyse our system by measuring the errors incurred through the processes of encoding, transformation and decoding of stimulus points. We proceed by choosing a point 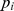 that represents a desired output, perform the necessary processing through the mapping to find the actual output 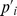 and then plot these on a 2D surface. This process is then repeated for a series of different 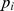. As a measure of error over a series of points we use the *expected absolute deviation*: 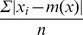
(8)where 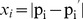, n is the number of errors and m(x) is the error mean: 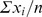. This error measure is usually normalised in the result plots by dividing by the field spacing. This was used as a performance metric for all the results but we also ran worst case examples too. The worst case results are not shown for space reasons but they always followed the same pattern as for expected absolute deviation but with increased magnitude.

### Linear transforms

Linear mappings are those in which the transform between 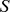 and 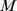 may be scaled or translated but are essentially linear in their axes. To better understand the effect of the overlapping processes involved we tested the encoding and decoding without transformation, thus providing a good test of the accuracy of the model by directly transmitting input to output. Ideally the output should be identical to the input, thus any difference between a single stimulus point, 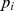, on 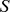, and resultant response point 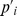, on 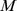 gives an error measure that can be used for assessing the quality of a mapping.

### Structure noise tolerance

It is possible that spatial noise may be present in the assemblage of a map of fields (both regular and unstructured). To examine this we performed a linear transform mapping with varying amounts of error in the field locations ranging from zero to half grid spacing.

### Non-linear transforms

We also examine the performance of our mapping scheme for non-linear spaces. In the linear transforms we effectively use discrete points as targets on 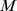 but it is possible that 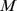 is also tiled with fields and therefore the encoding process must take account of the many possible fields that could define individual locations on 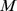. This is an extension from many-to-one structures to many-to-many. In order to manage this process each link is assigned a weight. These weights approximate the similarity in field locality such that if a field in 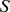 exactly maps onto a field in 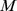 then the weight is one. If the centre point of a field in 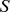 maps onto the edge of a field in 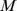 then the weight would tend to zero. A learning system could find these values but for our experiments the weights are approximated by projecting the centre point of the field in 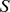 onto the map 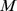 and then using the distance between the projected point and the centre point of the linked field. We then normalise the distance to the field radius and apply one of the activation functions described earlier. More formally, let 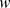 be the link weight, 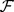 be the chosen activation function, 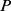 be the projected centre point in 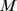, 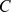 be the centre point of the field in 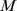 and r be its radius: 
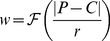
(9)


Applying these weights to the activation values of the stimulated fields in 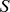 we can stimulate the fields in 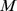 such that their signal is proportional to their relationship with their fields in 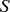. We can then decode the stimulus using vector averaging. Let 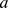 be the activation value of fields in 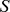, 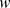 be the weight assigned to the link and 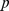 be the linked field in 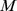: 
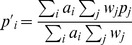
(10)


**Figure 10:**
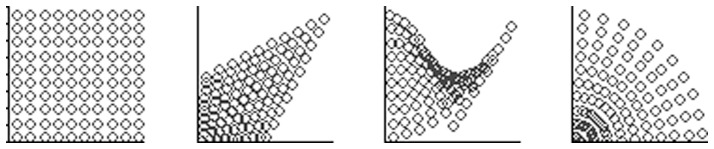
Space distortion. Distortion functions applied to a regular grid of points (left) using a compression function (centre left) a folding function (centre right) and a robot inspired transform (right).

We chose a range of fairly severe distortions as tests. First we tested the case of the target map being compressed into a smaller space than the source map. To examine this we created links between fields using the following transform: 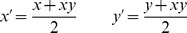
(11)where 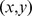 are the coordinates of a field on 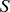 and 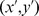 are the corresponding coordinates on 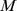. This gives a mapping that is identical along the diagonal but tends to compress into half space for off-diagonal elements, see figure 10 (centre left). Secondly, we looked at transformed spaces that produce folds. To create this effect we used the following transformation functions: 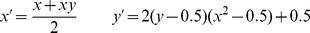
(12)


A third transform was based on a simplified robot arm design. It consists of a rectangular retina (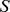) and a polar representation (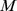) of a hand position that could move along radial and angular axes. The transformation from eye to hand was defined as: 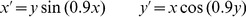
(13)


The above transformations are displayed graphically in figure 10.

### Estimating the number of fields and links

Our error values are quantified as a proportion of the regular distance between the fields. Using these values we can create a simple formula to calculate the distance required to achieve a given error on any map. Let 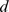 be the unknown distance between the fields, 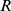 be the expected deviation we want to achieve and 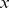 be the proportional error achieved by the chosen topology: 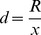
(14)


It is important to note that the only proportional value is 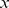. The values 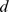 and 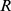 are defined according to the map space.

From the field distance we can approximate the number of fields 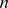 in a triangular map. Let 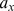 and 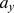 define the map area, 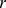 be a chosen radius, 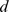 be the known distance between the fields and 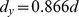 be the adjusted distance in the vertical axis: 
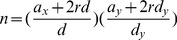
(15)


With a many to one mapping between 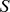 and 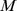, the number of links is equal to 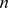. With a many to many mapping we can estimate the number of links by multiplying 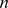 by the mean number of overlaps for the given topology, presuming that the topologies of 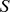 and 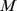 are the same.

### Vector reaching test

In order to examine how errors from overlap might affect a motor application we implemented a vector based reaching algorithm on a simulator for an iCub - a humanoid robot with an anatomical structure similar to that of a young child [28]. To keep the task simple and avoid problems with redundancy we chose a vector based algorithm that is able to reach targets within a reasonable frontal working area.

A 4 dimensional map of proprioceptive space for the first 4 joints in each arm on the iCub was created. The axes are in degrees of rotation and we call this space 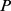. Each field in 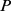 has a 4 dimensional centre point and is linked to a 3 dimensional point in a Cartesian gaze space that's relative to the base of the torso and is measured in meters. The gaze point represents the position of the hand experienced when the proprioceptive feedback matches the centre of the field in 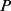.

Each of these fields also contains a map that describes the change in gaze space that the hand experiences when a motor command is applied over a small distance. This map, 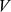, contains fields with a 4 dimensional centre point representing a motor command and has a 3 dimensional vector that represents the change in gaze space caused by the application of motor command. Through a hand regard process of making small movements from the field's centre point the motor space is learned by populating map 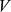 with the results.

When the maps have been learned sufficiently we can attempt to move the hand from any reachable point to another using vector averaging. First we derive a vector 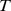 by subtracting the gaze point for the hand from the gaze point for the target. Then we activate the fields in 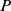 according to the current proprioceptive values using a Gaussian response function. For each active field 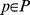 we activate the fields 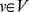, also with a Gaussian function, using the angle between the 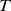 and the gaze vector 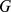 in the 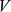 fields. Instead of a field radius we use a maximum angle 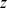 and only include fields within that angle. We can then find the response function: 
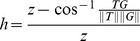
(16)


Using these activation values we can perform a weighted vector averaging to estimate the local motor command that will move the hand towards the target. Let 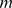 be the motor command, 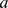 be the activation value of field 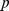, 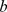 be the activation value of field 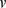 and 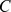 be the motor command in field 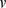: 
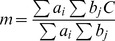
(17)


By regularly recalculating the motor vector the hand will eventually reach the target. Measuring the distance travelled by the hand throughout the move produces a measure for the quality of the reach action. We begin from a fixed start position and move through a fixed set of target points. We then compare the distances against the ideal straight line distances to establish the quality of 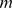. The test was performed multiple times using different field radii in map 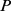.

### Position based reaching

The vector based reaching experiment demonstrates the use of linear mappings and so another test is needed for non-linear transformation features. We do not address the full robot solution here; we avoid the issue of redundancy and focus on the mapping between visual location and positional reach information.

We generated two maps: a 3 dimensional gaze map and a 4 dimensional proprioceptive arm map using the spaces described in the last section. We then apply a simple learning algorithm that create links between the maps to describe the positional transformation. Learning was performed over a period of 10 minutes by moving the arms randomly whilst monitoring the gaze point of the hand. As the hand moved through fields in the gaze space then links were made to the currently activated arm fields such that the value of link was set to match the activation in the proprioceptive arm field. This value is only set when the activation of the gaze field is the highest it has experienced so far. After learning the links we then estimate the arm configuration needed to reach a point in gaze space using the weighted vector averaging equation (10).

## Results

### Linear transforms

#### Error patterns

**Figure 11:**
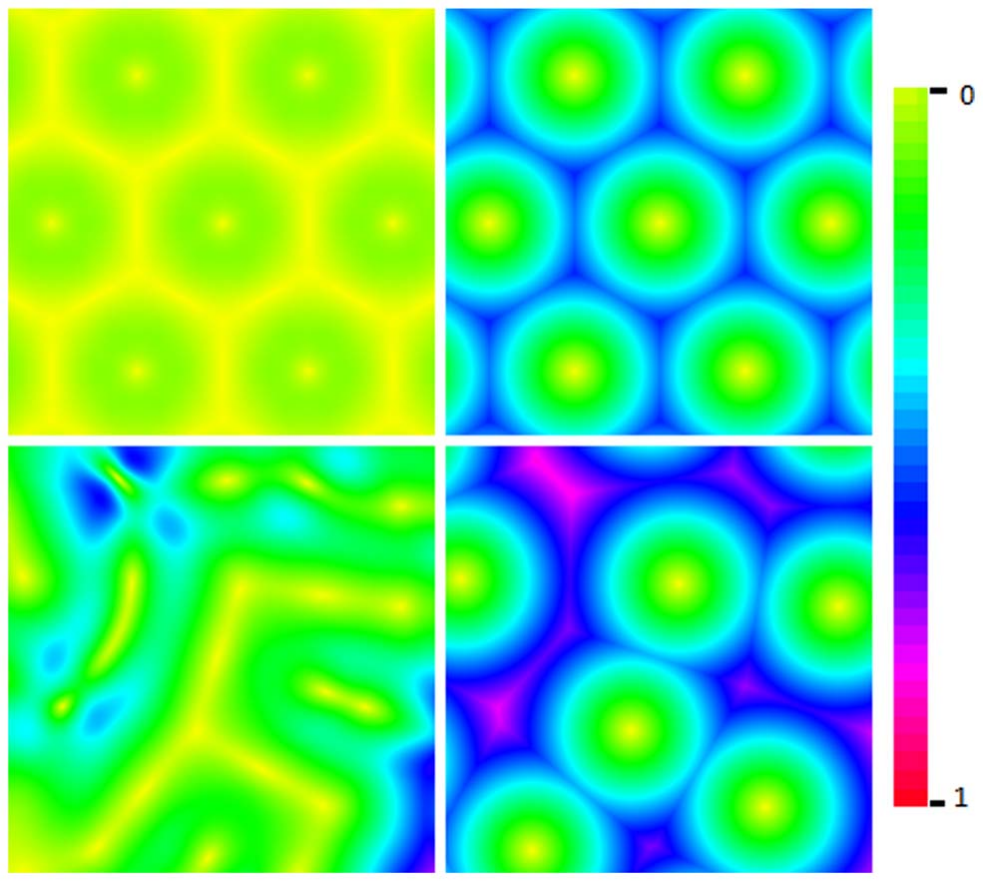
Benefits of overlap. Error map depicting triangular (top row) and random (bottom row) topologies. The left column uses vector averaging and the right column is a single field response. Each pixel on the map is used as a stimulus point and the distance between the result and original point is denoted by the colour of the pixel. Errors range from 0 to 1 field radius.

**Figure 12:**
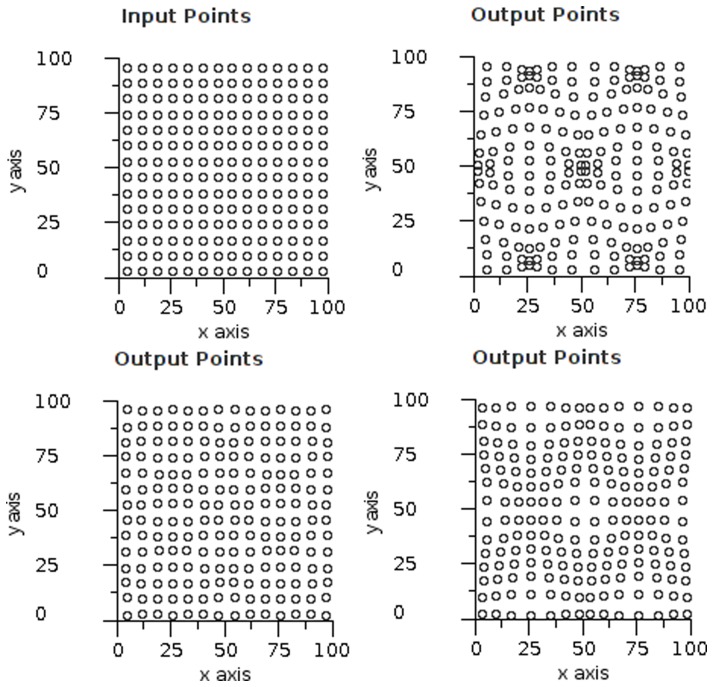
Cosine overlap visualisation. Input test data and output arrays from experimental software. Each point from the input grid (top left) is encoded using a cosine function and decoded using vector averaging. The axes are scaled such that 50 units  =  one grid spacing. The results show a field radius of 1.0 (top right), 1.1 (bottom left) and 1.2 (bottom right).

**Figure 13:**
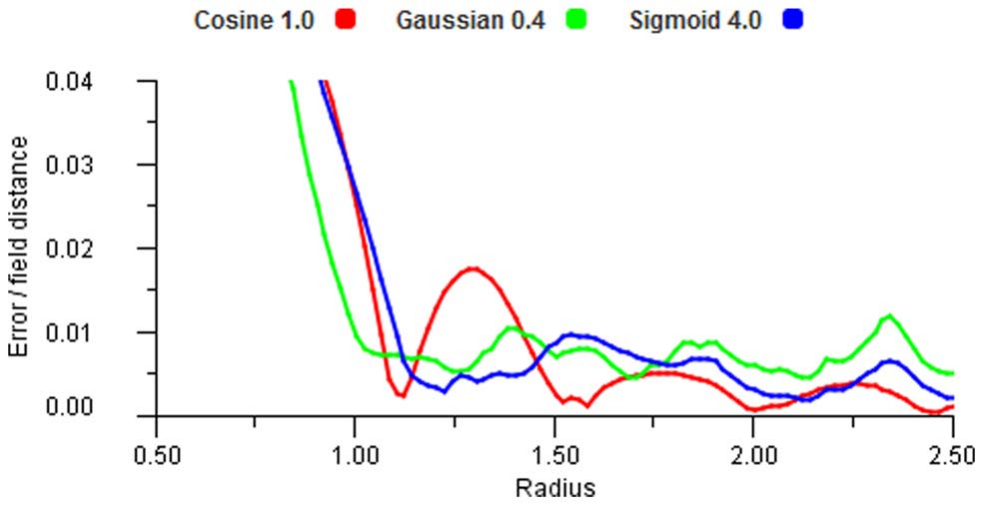
Error plot for a linear transform. Both S and M maps have identical triangular structure and a one to one transformation space.

**Figure 14:**
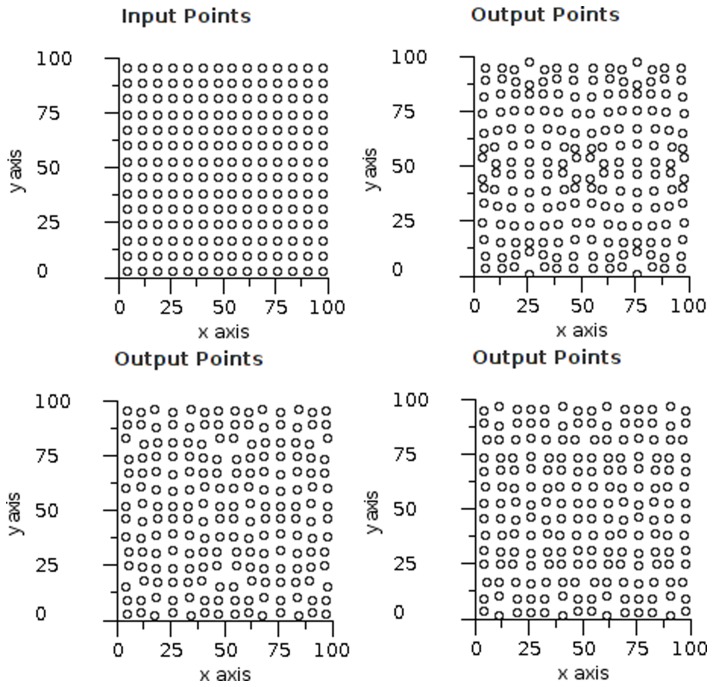
Gaussian overlap visualisation. Input test data and output arrays from experimental software. Each point from the input grid (top left) is encoded using a Gaussian function and decoded using vector averaging. The axes are scaled such that 50 units  =  one grid spacing. The results show a field radius of 1.0 (top right), 1.1 (bottom left) and 1.2 (bottom right).

The simplest decoding of an input set is to use the “single neuron” response from the most active field. This is seen in figure 11 in the right column. The error clearly increases as the stimulus moves away from the field centres and the maximum error is at the equidistant points between fields on a triangular grid. The left column of figure 11 shows vector averaging and it is noticeable that this gives zero error in the equidistant regions in the triangular structure (top left). We observe how errors can increase in sparse regions of an unstructured map, and how the use of the overlap by vector averaging markedly improves the results. Figure 12 shows a set of input points forming a uniform grid and three different outputs for increasing field radii. An ideal result would show the output points to be in exactly the same positions as the input points. It is important to note that each point is computed separately — they are only combined in the display to give a visualisation of the error pattern. For a radius of 1.0, in figure 12 (top right), the underlying field structure is evident in the error distribution (the field centres are at the cluster points), but this disappears for 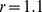 which gives a very good match to the input map. Perhaps surprisingly the error increases at 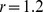, as can be seen in figure 12, (bottom right). This can be explained by figure 13 which shows the total error over all points (as expected absolute deviation/field spacing) against increasing radii. There is a noticeable oscillation effect, which is seen in many of our results, due to the subtly changing overlap patterns as the radii change. From this plot it can be seen that 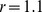 gives a lower error result than either 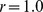 or 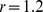 for the cosine function. The experiment was repeated for a Gaussian encoding and figure 14 shows the results. This case shows similar patterns but is less revealing of the underlying structure. Although error distributions are visible to the eye in these displays it should be noted that they are actually very small individual displacements and the total error measure is the important indicator. All the error values are very low for 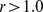 and generally continue to reduce as 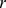 increases. Figure 13 also shows that Gaussian, cosine and sigmoid functions tend to produce largely similar results at large radii.

#### Trilateration and noise tolerance

**Figure 15:**
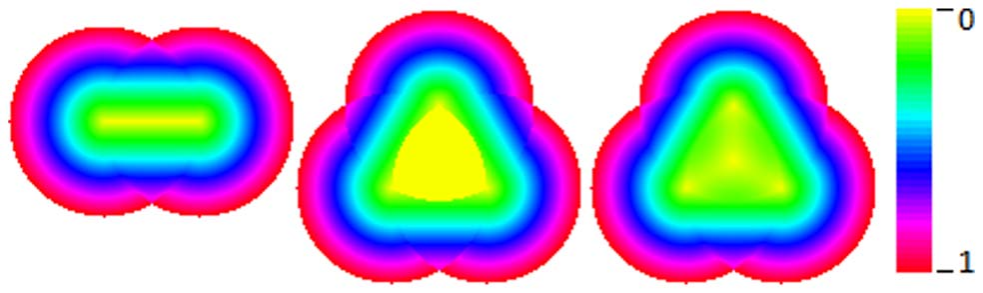
Small sample map. Error map depicting 2 fields with trilateration decoding (left), 3 field trilateration (centre) and 3 fields vector average decoding (right). The encoding function is linear for all cases. Each pixel on the map is used as a stimulus point and the distance between the result and original point is denoted by the colour of the pixel. Errors range from 0 to 1 field radius.

In order to observe the effects of trilateration we used a linear falloff response function and applied this to just two and three fields. Figure 15 shows that for the case of overlap between two fields we get zero error along the line joining the field centres and for a three field overlap there is also complete accuracy within the convex area defined by the centres. By comparison, the decoding by vector averaging shows full accuracy only at the field centres and at their equidistant centre.

**Figure 16:**
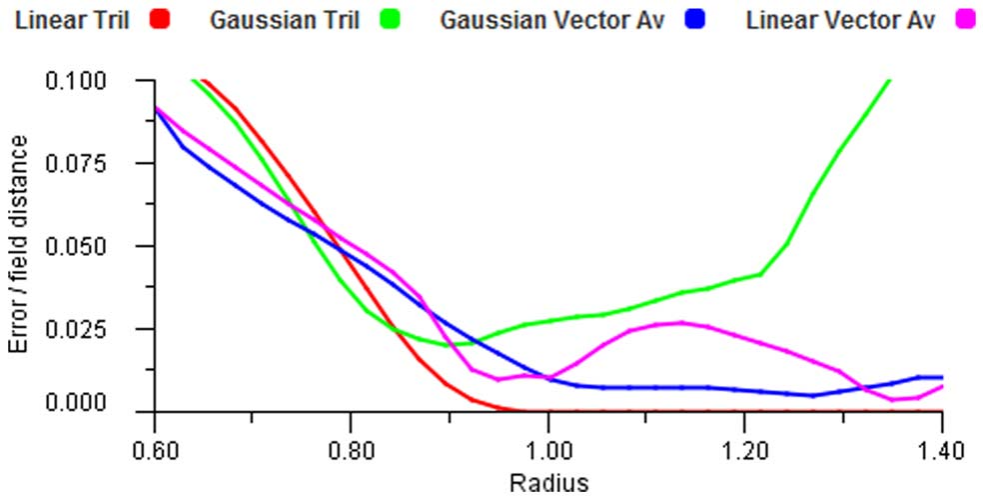
Accuracy without noise. Error plot for increasing field radius showing the difference between vector average and trilateration methods. The Gaussian responses have a coefficient of 0.4.

However, trilateration depends upon the signals being proportionate to distance and therefore we can only expect high accuracy for linear response functions. If we use smoother response functions like Gaussian or cosine functions, [29], then the errors can be reduced somewhat by performing the trilateration calculation three times, changing the order of the points each time and then averaging the results. Figure 16, seen earlier, shows a precision comparison between such trigonometric based trilateration and vector average decoding. The trigonometric version is very accurate once all areas are covered by three fields, but only for the linear coding function. The Gaussian response with vector averaging performs well otherwise.

**Figure 17:**
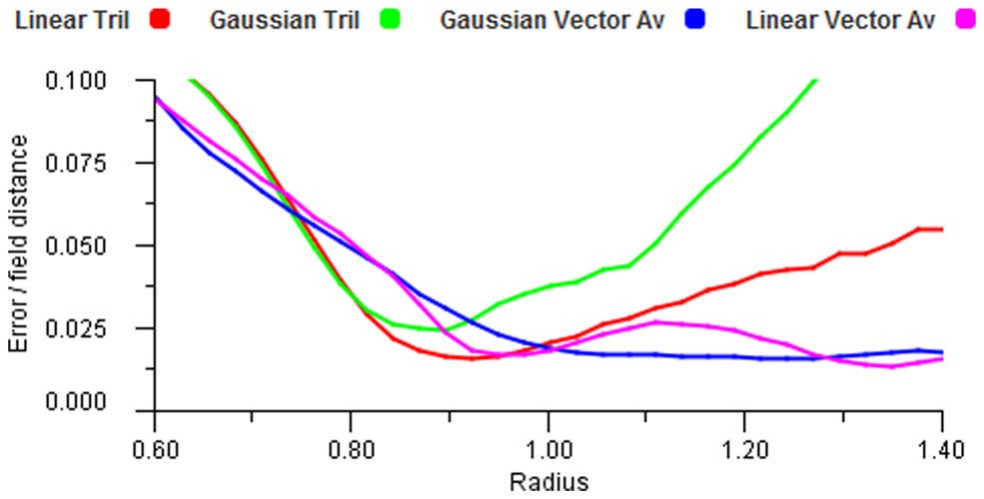
Accuracy with noise. Error plot for increasing field radius showing the effect of noise on the vector average and trilateration methods. The Gaussian responses have a coefficient of 0.4 and the noise coefficient 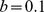.

When noise is introduced this comparison is accentuated. Figure 17 shows the results when noise is added to active fields. It is clear from these figures that trilateration is of value only in the specific case of linear response functions. Despite the high accuracy possible (as seen in global localisation with GPS [26]) its use is not feasible for functions compatible with biological situations. We notice that vector averaging improves in accuracy with increasing input contributions, i.e. larger 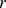. Thus, the accuracy of the central area in figure 15 (right) will improve further if more than three fields are included.

Vector averaging gives a reducing error trend, particularly with Gaussian encoding and tolerates noise very well.

#### Response functions

**Figure 18:**
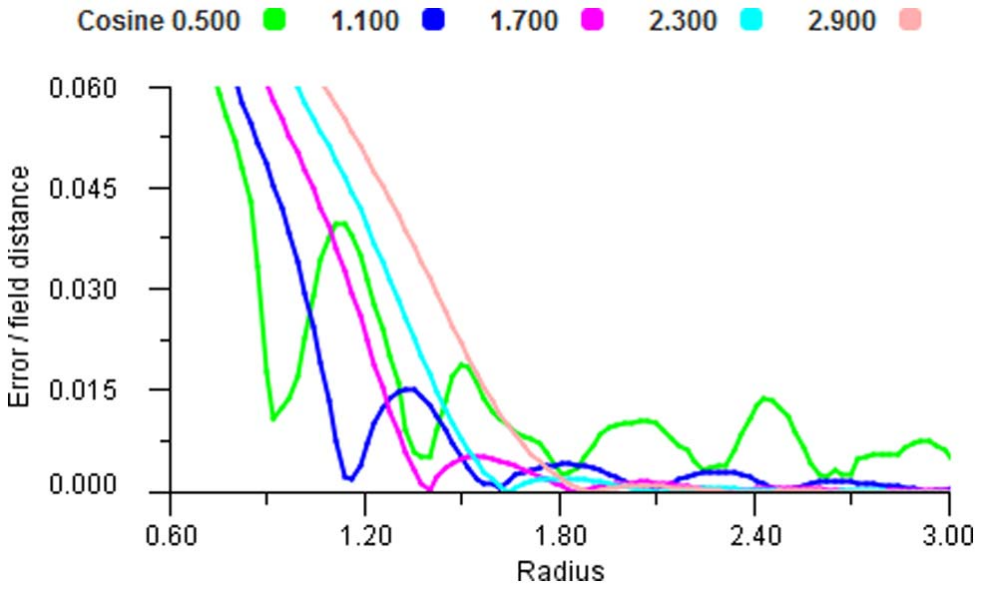
Cosine performance. Error plot for a range of cosine encoders using vector average decoding tested over a range of radii.

**Figure 19:**
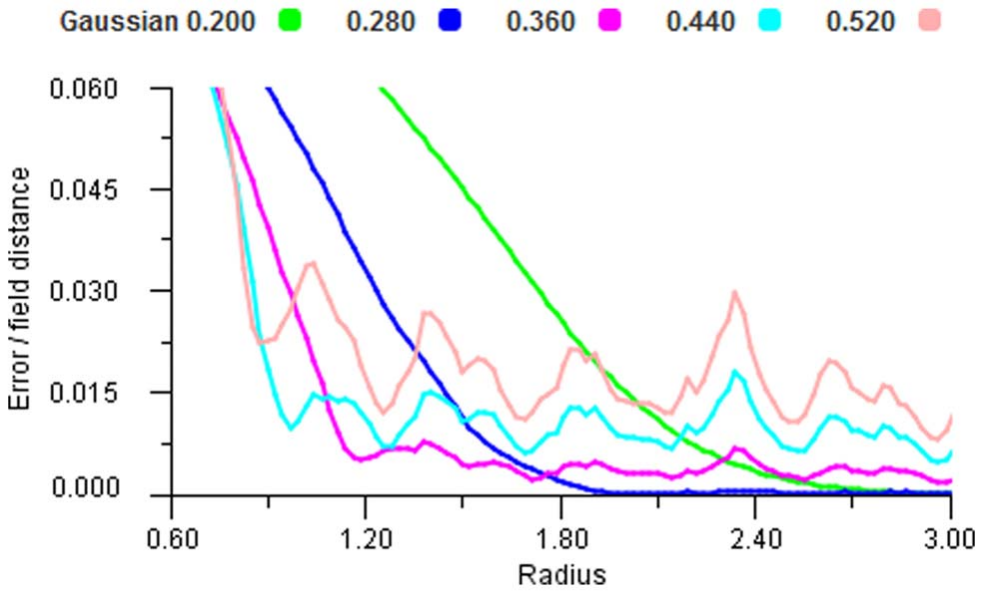
Gaussian performance. Error plot for a range of Gaussian encoders using vector average decoding tested over a range of radii.

**Figure 20:**
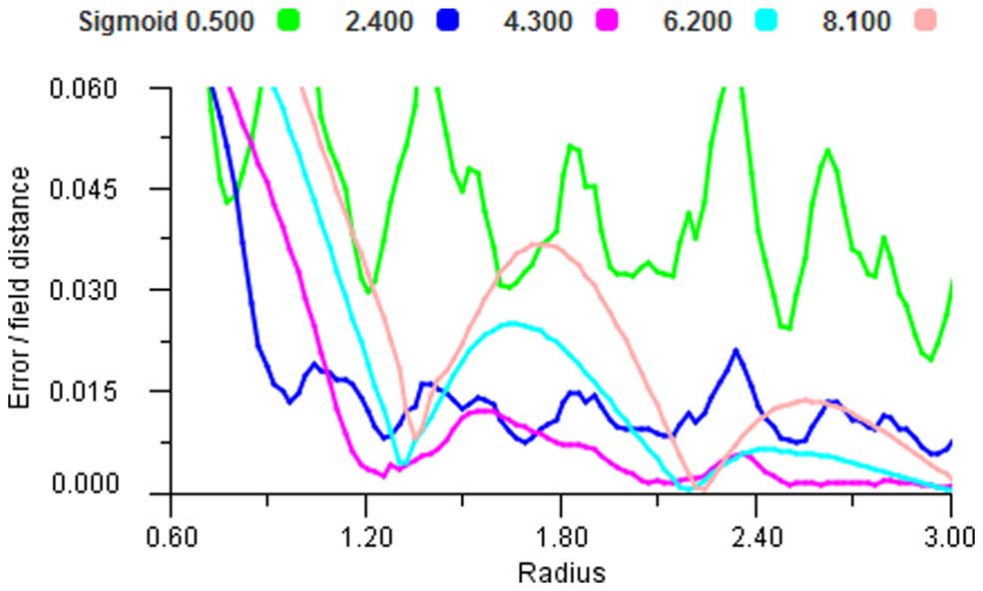
Sigmoid performance. Error plot for a range of Sigmoid encoders using vector average decoding tested over a range of radii.

**Figure 21:**
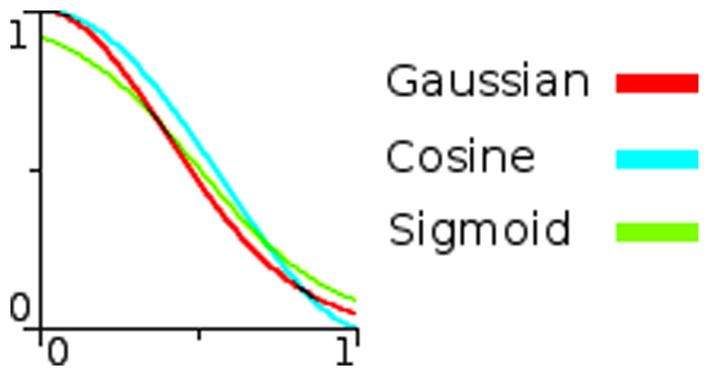
Response curve comparison. Response curves for Cosine 0.8, Gaussian 0.4 and Sigmoid 2.4. The input is along the abscissa and the response is along the ordinate.

In order to examine the behaviour of different non-linear response functions we took a large set of sample points and ran these through the mapping for increasing radii. Figures 18, 19 and 20 show that all functions can display the oscillating effect but there are coefficient values for each function that give good performance. We selected the most effective coefficients (Cosine 0.8, Gaussian 0.4 and Sigmoid 2.4) and used these for the remainder of the experiments. It is worth noting that these best selected coefficients for the encoding functions produce very similar shapes, see figure 21.

#### Structure and boundary effects

**Figure 22:**
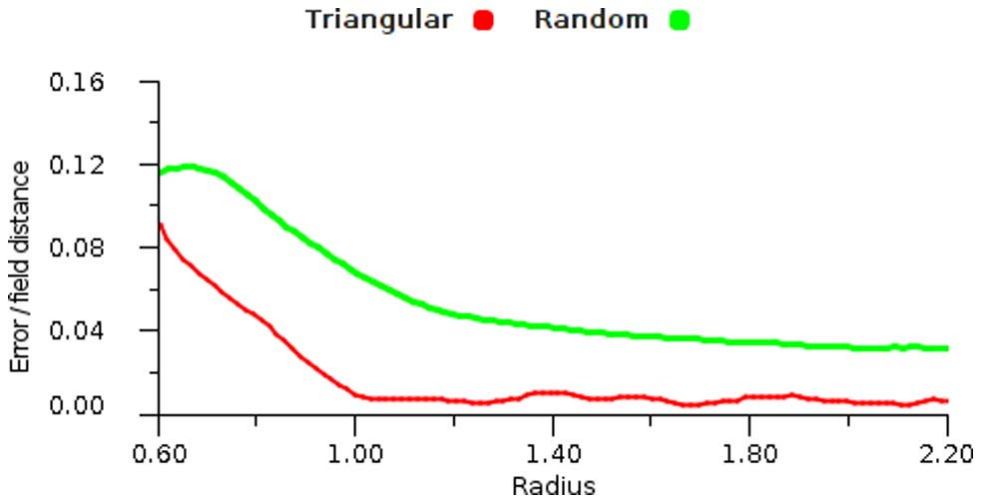
Topology structure comparison. Error results for a Gaussian response function with a coefficient of 0.4 and a vector average decoding. The test is performed with increasing radius using triangular and random topologies.

We tested the effect of structured field placement using a triangular grid and an unstructured set of fields (random placement) using the same data set and a range of encodings. Figure 22 shows the results and for the regular grid the error rates decline quickly towards approximately 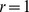. For irregular fields the errors are higher and we found that all the different response functions gave very similar results.

**Figure 23:**
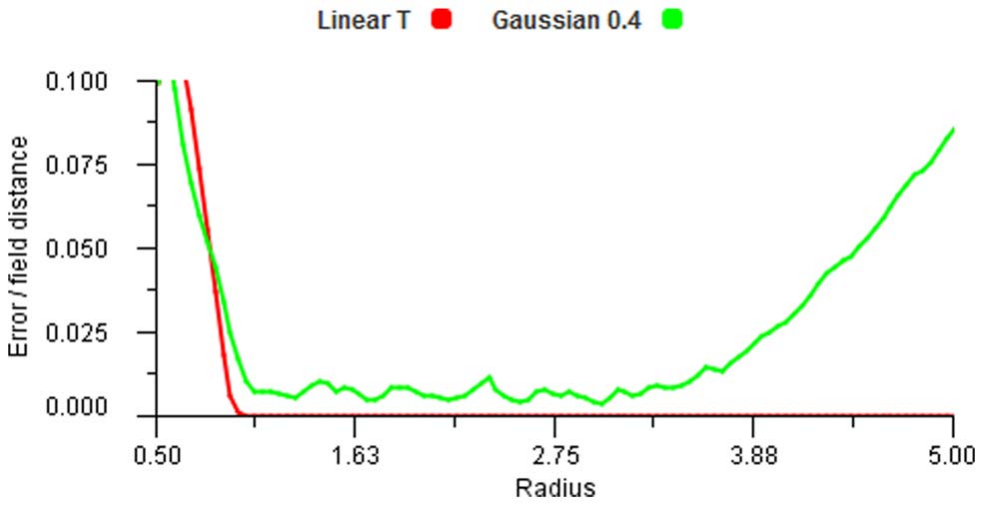
Border effects. Encoding accuracy graph showing the effect of large overlaps at the borders, for 10×10 array.

When overlaps become large (e.g. 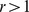) increasing error effects will be noticed near the edges of the maps. This happens because fields in the border region receive fewer contributions from the side nearer to the edge. Figure 23 shows the errors (for a 10 by 10 array of fields) increase dramatically as increasing overlap encroaches on the boundary, thus reducing the effective working area. For interest, we notice that trigonometric trilateration does not suffer from this problem.

#### Field and link density

Using the results provided by figures 18, 19 and 20 we can calculate the distance between the fields and therefore estimate the field and link count needed to achieve a given error using the equations 14 and 15.

The performance of an algorithm that uses the map will potentially be effected by the number of fields in the map and the number of fields and links included in encoding and decoding. Reducing encoding errors by increasing the overlap can reduce the number of fields needed to cover an area but it increases the number of links in the map and the number of fields used in an encoding.

**Figure 24:**
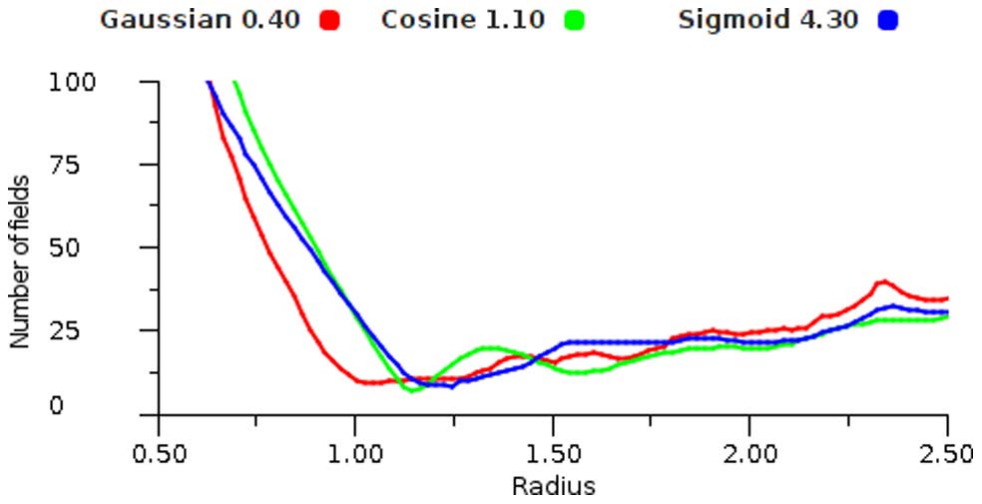
Field count versus error. The number of fields required to provide an error of less than 1 percent of the map's area, plotted against field radius, using a range of functions on a regular triangular grid.

**Figure 25:**
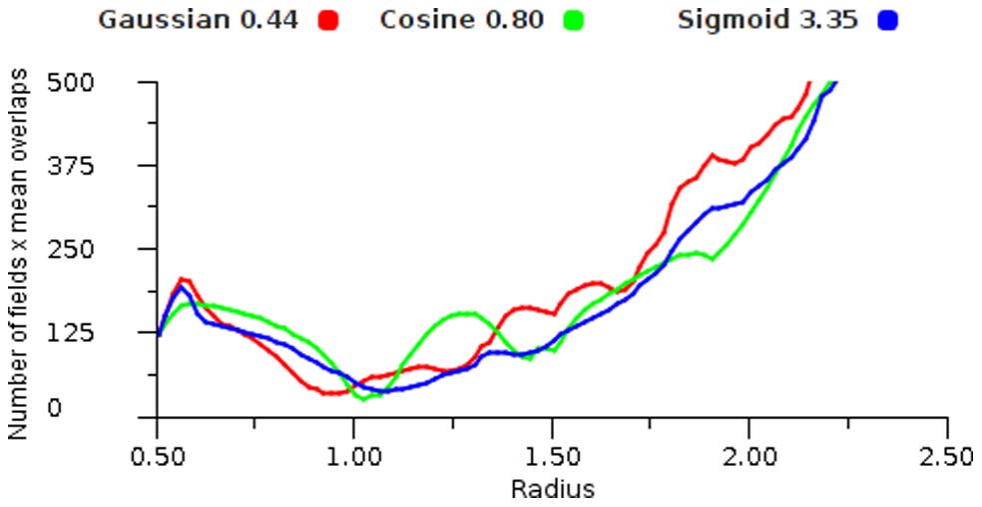
Link count versus error. Combined mean overlap and field counts required to provide an expected deviation of 1 percent of the map's area. This approximates the number of links between maps of similar density.

Figure 24 plots the total number of fields required for an expected deviation of less than 1 percent of the map area, using increasing field radii. Various values for the response functions were tested and we selected the values that achieved the smallest number of fields at some point in the radius range. We see that the number of fields actually increases as the overlap increases over 1 radius because extra fields are needed at the borders of the area. Figure 25 multiplies the number of fields required by the mean number of overlaps in the given topology to provide a simple estimation of the number of potential links between two similar maps with a linear transform.

#### Structural noise tolerance

**Figure 26:**
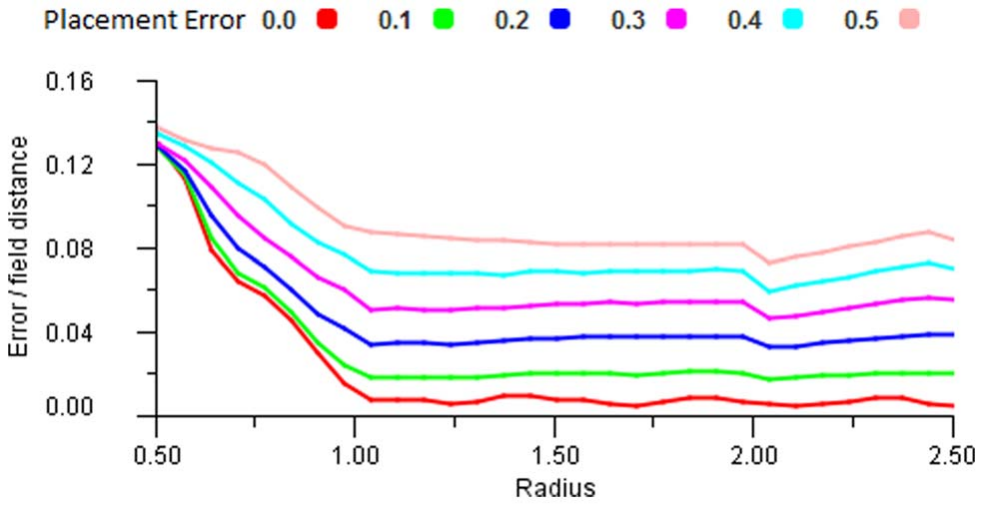
Transformation with placement error. The accurracy of a triangular structure with various levels of error added to the grid placement of the fields. The compression transform is used and the response and link functions are Gausian (0.4).

Structural noise can be defined as the errors due to field centres being displaced off-grid in a structured lattice. As fields become increasingly displaced so they move from structured to unstructured. Looking at figure 26 we can see that some structural noise can be tolerated but we note that at 0.5 placement error the resulting map would no longer resemble a triangular structure; it would appear random, and these results confirm those of figure 22 that a structured grid seems beneficial.

**Figure 27:**
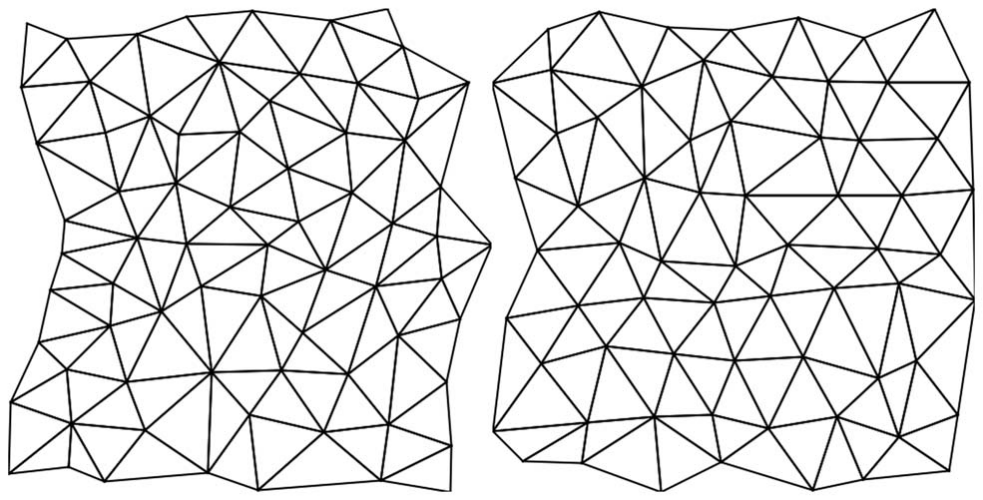
Real and model placement error structures. Triangulation of an array of cat retinal cells (left) and generated fields (right) for spatial comparison. The generated set has the same standard deviation (approximately 0.21) and was produced with a placement error of 0.35. The neuron array is digitised from figure 5 in [30]

To compare the results with real neuron arrays we digitised the locations of the cell centres from a sample of a cat retinal structure (figure 5 in [30]) and then measured the variance from the ideal regular structure by triangulating the points and performing a standard deviation measurement on the distances apart. This gave gave a standard deviation of 0.22 for the cat retina. By performing the same test on generated structures with various degrees of error placement we looked for a result with a similar standard deviation. Figure 27 compares the triangulation for the real neuron case (left) and a generated set with matching parameters (right). The randomly generated case was analysed and gave a placement error of 0.35 which is a reasonable compromise between exact grid placement and random locations. It is interesting that the biological structure, while unable to produce a perfect lattice nonetheless gives a very even and effective coverage.

### Non-linear transforms

**Figure 28:**
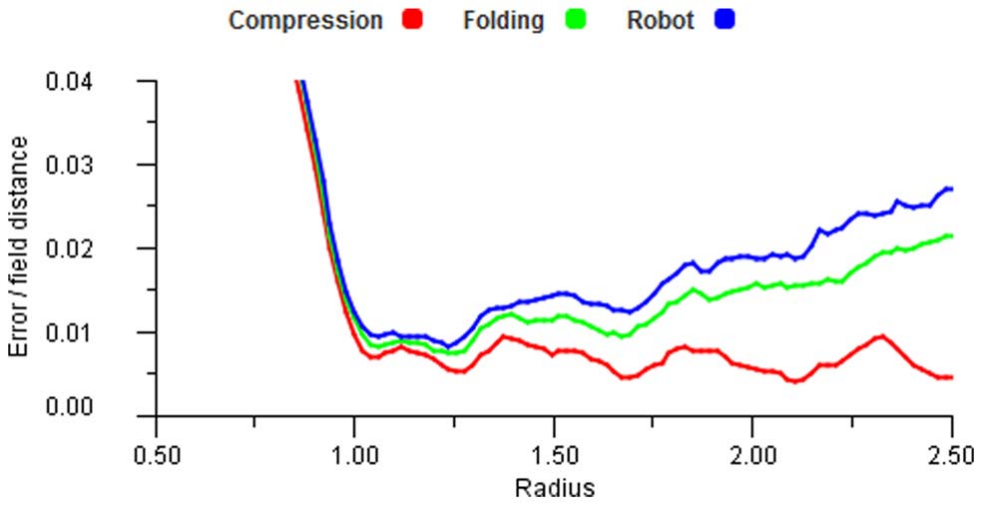
Transformation errors. Error plots for the different distortion transforms with increasing radii. A Gaussian (0.4) response function was used for encoding.

**Figure 29:**
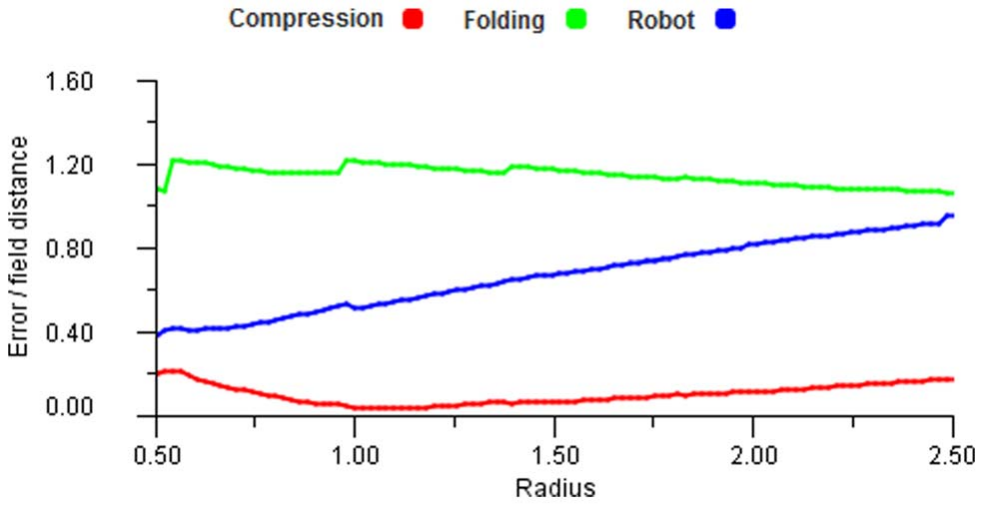
Reverse transformation errors. Error plots for the different distortion transforms processed in reverse. A Gaussian (0.4) response function was used for encoding.

**Figure 30:**
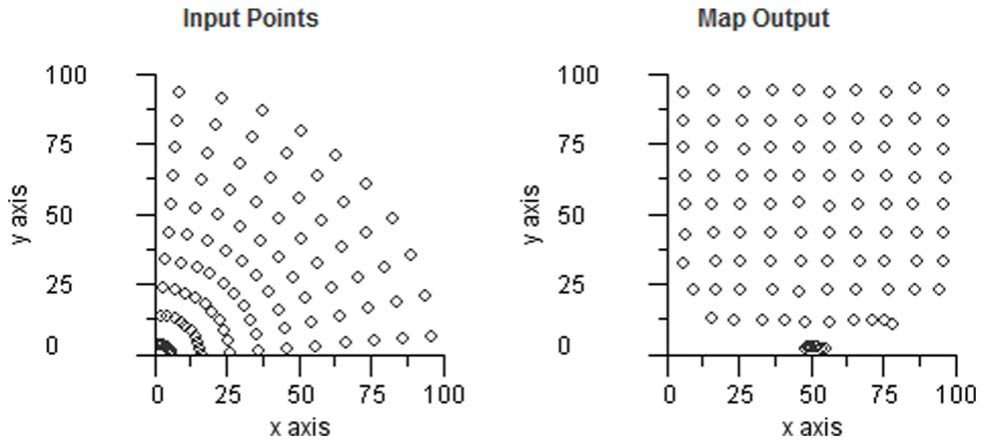
Transformation information loss. A reverse transform using the robot distortion space, i.e. from the points in hand space (left) to eye space (right). The ideal result would be a regular grid of points. The errors in the bottom rows of the square grid are caused by the distortion squeezing points into the polar centre in the bottom left corner.

Using the three non-linear transforms for the mapping (equations 11, 12 and 13), we tested a range of response functions and found the results to be comparable. The most significant influence on the errors is the degree of distortion of the space itself. Figure 28 shows the results for a Gaussian (0.4) function processed through the three transformed spaces as shown in figure 10. Figure 29 shows the results of the same process but with the reverse transform, i.e. mapping from the distorted space to the regular grid. The reverse cases produce even more severe distortions and this is clearly reflected in the error results. These results indicate that larger fields with many overlaps do not increase accuracy for non-linear regions because they incorporate more contributions from fields that obscure the detail of the distortion. This suggests that there is little to be gained by having more overlapping than that provided by 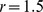. Figure 30 shows the reverse mapping for the robot example and the result can be seen to be very acceptable for regions with well spaced fields — the errors arise where the input fields are compressed into a small area.

**Figure 31:**
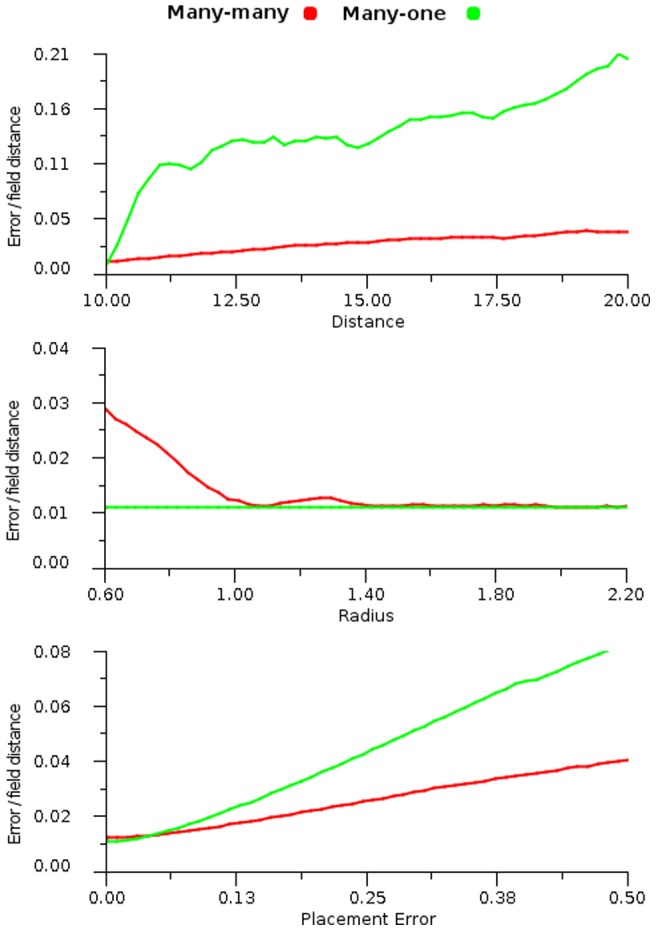
Transformation error comparisons. Error plots of cross modal maps using a compression transformation function and varying: distance (top), radius (middle) and placement error (bottom). The results compare the behaviours of the many-to-one and the weighted link many-to-many method. In each graph the transmitting map 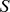 is fixed at 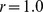, distance spacing 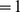 and zero placement error, whilst the receiving map is adjusted as described in the abscissa.

As a comparison of many-to-one mappings with many-to-many we ran experiments with a fixed field arrangement for 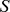 while varying the parameters of 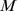. Figure 31 shows the results for spacing distance, field radius and field centre error when transforming points from one map to another with the compression transform. The many-to-one structure provides the best possible result if the centre point of the field in 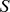 precisely transforms to the center point of the linked field in 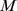. When this is not the case then using a weighted, many to many process can produce better results.

### Robot reaching

**Figure 32:**
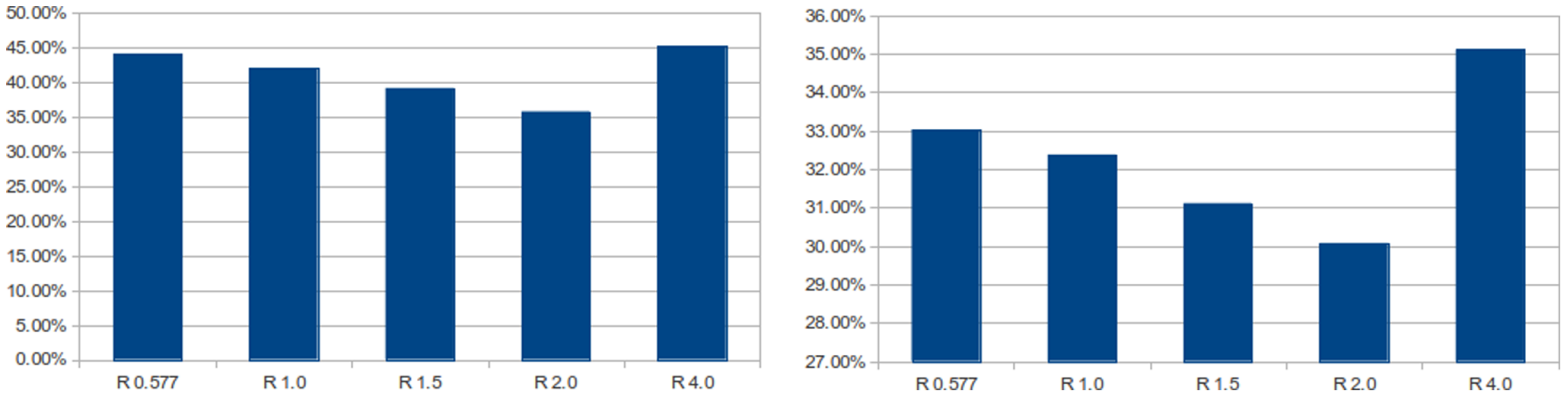
Vector based reaching performance. Shows the average percentage of extra distance travelled over the ideal distance whilst moving the hand of the robot through a set of points in the gaze space. Each bar demonstrates the change in performance with increasing field radius 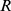. The experiment was performed with random (left) and rectangular based grid (right) topologies.

Figure 32 shows the effect of field radius on the vector based reaching algorithm. We see that, as predicted by figures 22 and 23, the accuracy of the results improves as the radius increases up to a point. Our algorithm does not compensate for borders around the map and so we expect errors to dramatically increase when the radius is so big that samples at the edges become imbalanced. We see exactly this effect at a radius of 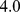. We note that in figure 22 the irregular topology provides an argument for using radii of more than 1.0 as opposed to the triangular topology which provides little benefit after 1.0. The results in figure 32 are consistent with this: error reduction for 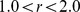 is much less for the grid based case. It is also worth noting that this demonstrates the applicability of the results when using more than 2 dimensions.

**Figure 33:**
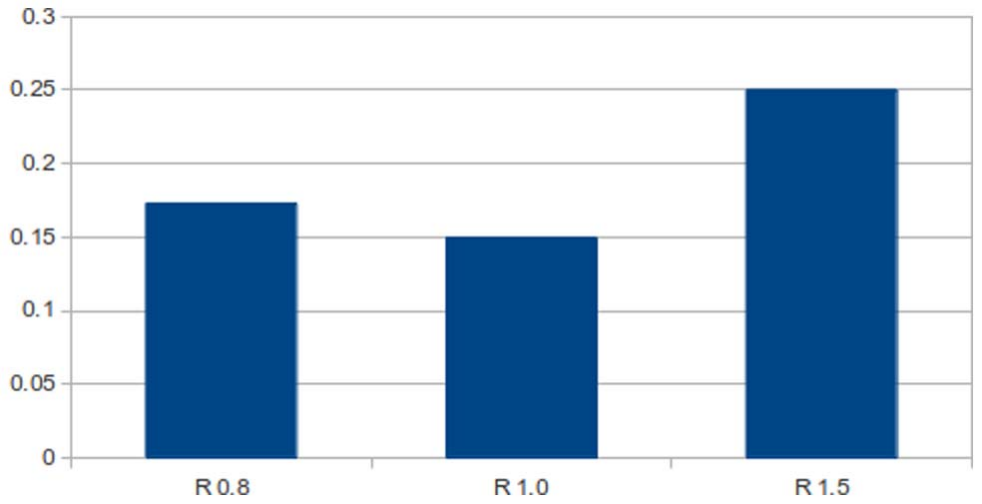
Position based reaching performance. Shows the average reach error experienced with field radii 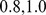 and 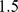. The experiment was performed with a rectangular based grid topology.

Figure 33 shows the effect of field radius using a non-linear transformation for position based reaching. We see that after a radius of 1.0 that accuracy of the reach diminishes. Looking back at figure 28 we note that errors are likely to increase after 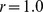 for this kind of distortion. We also note that because the arm has redundant degrees of freedom some deterioration in vector averaging could be expected. This could be remedied by filtering the activations but this is beyond the scope of this paper. Even so, the relationship in the results between accuracy and overlap remain true to the earlier results.

## Discussion

Topographic maps are ubiquitous in the brain [14] but making long connections between such maps is costly [31] so some method of reducing the number of long distance connections, whilst maintaining a high enough quality of information transmission, is necessary for large neural systems. Various ideas, such as small world networks [32], have been suggested for this problem and our results show that overlapping fields can also offer solutions in reducing the required number of links between maps to achieve a given spatial performance.

We focus on the question of how overlap affects locational accuracy in topographic arrays and on transformational mappings between arrays. That is, given a stimulus point on a map 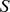 and a transformed map 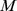, what influences the accuracy of the location of the response on 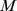
? Through software simulation we explored the behaviour of circular overlapping fields, examining the variables and comparing differing stimulus response functions and decoding methods. We found there are two main variables: field radius, which determines degree of overlap; and field structure, ranging from strict lattice formation to random placement.

The results in figure 11 show a clear benefit for using overlap where much greater overall accuracy is achieved for encoding. Specifically, when used with vector averaging, overlap shows greatest improvement in the areas of highest error in the single response map. In considering any detrimental effects we looked at the cost of decoding in terms of complexity against varying levels of overlap. Complexity reduced as the radii increased until it reached 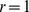, the common spacing between the fields. We also note that very large overlaps, when used with vector averaging, reduce the accuracy around the edges of the maps. A first summary of these points would be that some overlap provides increased accuracy but very large overlaps are detrimental.

Considering field structure, we find a regular grid has advantages over an unstructured array of fields. Structural noise, that is, variation away from the ideal triangular lattice, was seen to introduce error (figures 11 and 22) and this is particularly noticeable for maps with little or no overlap. For such (small) fields, relatively large errors occur as the grid lattice breaks down and approaches random field placements. Regarding unstructured field distribution, independent field generation ensures that the maps are accurately shaped by early experience. However when the maps are fully populated it is possible that the differences become marginal. We observe that grid-based placement can be useful in situations where some external structure must be taken into account. Genetic constraints on field size and placement can be built into such structures. Of course, in many cases the target map will be generated from experience and will relate to irregular spaces that can not be grid-based beforehand.

The location of a stimulus can be encoded in various ways and we tested response functions that are commonly found in neural network studies (step, Gaussian and sigmoid), and functions that are interesting for their trigonometric properties (uniform, linear and cosine). We found that the non-linear functions, cosine, Gaussian and sigmoid, were the most effective, with the Gaussian being slightly better behaved over all situations. However, the functions are very similar for the values we eventually selected as best: 0.2, 0.4, and 0.8 for the cosine, Gaussian and sigmoid respectively. It seems that the fine detail of the curve shape is not highly critical.

For the decoding of spatial locations we experimented with two decoding methods: trilateration and vector averaging. Trilateration was very accurate with linear encoding and strictly regular grids but without these two conditions performance is consistently worse than vector averaging. Non-linear encoding functions have the effect of smoothing errors and this increases with contributions from more fields, hence large overlap is desirable and very effective with vector averaging. This is confirmed by work on averaging and combinations of estimators [33]. Thus the combination of vector averaging decoding with non-linear encoding functions gives good tolerance to noise, both signal noise and structural error.

The tests on distorted transforms used fairly extreme cases in order to examine worst case trends. There is a clear relation between deterioration of the mapping and the severity of the distortion; regions of folding and tight clustering produce much worse errors than smoothly varying non-linear distortions. From this it follows that smaller field radii should be used in regions of severe distortion because otherwise the decoding averaging operates over increasingly incompatible contributions and so errors increase considerably. In some earlier experiments we varied the field sizes, within a map, according to the degree of local non-linearity found during learning [34]. For an uncovered stimulus the learning mechanism causes a new field to be created precisely centred on the stimulus, with the size of the field determined to maximally fill the gap between the nearby fields.

From the results we can suggest an optimum degree of overlap. Most of the results for linear or slowly varying non-linear transforms suggest that the degree of overlap for best performance should be in the range 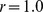 to 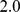. Overlaps involving many fields (e.g. 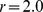) give good tolerance to placement error. However for very non-linear transforms such large values cause detail to be lost and 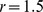 was found to be an upper limit. We consider 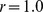 to 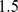 to be an appropriate range for most situations, and this gives areas of 3 fold to 7 fold overlap in the field structure.

We can summarise our findings as follows:

- Overlapping fields can provide distinct benefits in reducing the number of connections between topographic arrays when representing complex spatial transforms. For a representational accuracy of 1% in an array of size 100 units square, then only 50 fields (and hence 50 to 200 connections depending on the link topology) are more than sufficient if the transformation is not too severe.
- Very large degrees of overlap become increasingly detrimental as non-linearities in the mapping increase. This is because fine detail can be lost and resolution reduces.
- The choice of encoding function is not critical. More important is the coefficient used; the main criterion being a smooth falloff over the whole field radius.
- The size (radii) of fields should be larger for noisy systems, as increased overlap takes in more contributions and thus increases accuracy.
- Conversely, field sizes should be small for maps in regions of severe distortion or rapid structural change. The details of such severe non-linearities are better captured by increased density of fields in such regions rather than being lost in large overlaps.
- For a given error level the number of fields required, and potentially the number of links between maps, reduces as overlap increases up to 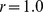. Generally there is little advantage in field sizes 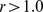 as there will be redundancy in the number of links.
- Regarding the incremental learning of mappings, the length of learning is proportional to the number of links that need to be established for total coverage. To reduce the density of links, larger fields can be traded off against fewer fields (i.e. by increased spacing). This speed up in learning will reduce accuracy but could be an important developmental technique; when a gross but complete map has been established then accuracy can be increased (with additional smaller fields) in areas of special interest.
- Another useful feature for learning is that an incomplete mapping can be used while it is being learned. As soon as a link has been established it can be used, and so a developing system may show some heavily used regions together with under-mapped regions, according to experience.
- Considering all the results we find that 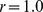 to 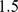 gives sufficient degree of overlap to provide effective mappings for many sensory-motor applications in robotics. The lower end of this range is appropriate for regular triangular grid spacing and non-linear transforms while the higher end can reduce density and compensate for grid irregularities and noise.

## Conclusions

We approached the issue of overlap from an engineering perspective; we have previously used overlapping fields to produce successful sensory-motor mappings in robotic systems [10] and we wish to understand the reasons for their performance and the parameters for designing such mechanisms. From this perspective the findings in the above section give some general conclusions about our abstract model. We can now turn to the neuroscience literature to look for any relevant studies that might complement our results from a biological viewpoint.

Examples of overlapping field effects can be found extensively in the brain; for example, in the retina [35], in the superior colliculus [8], and in sensory and motor cortical areas [36]. The fields are not physical entities like synapses but are formed through the cellular organisation. Hence, in the retina [37] the relationship between the (physical) dendritic structure and the (effective) receptive field structure can vary considerably, but this is not reflected in the degree of field overlap, which is more consistent.

Lehky and Sereno [38] have examined the phenomenon of overlap in the context of population coding, however, their study focused on intrinsic spatial representations whereas our maps are strongly grounded in the extrinsic coordinates of the sensory-motor systems. Also, many papers consider a given field size and report results against variable spacing between fields, whereas we fix the field centres and vary the field radii; this means conversion calculations may be required when comparing results.

Regarding optimum overlap, we find considerable agreement with our estimates for field size and spacing. For example, [39] report retinal ganglion cells as sharing about 40% between pairs, with 3-fold overlap and 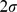 spacing between field centres. This refers to the practice of fitting a Gaussian to a field where the field size is defined as 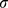, the standard deviation of the Gaussian. To compare this with our result we notice that our Gaussian encoding function terminates at about 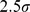. This means when 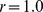 then the spacing between centres is 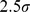. Increasing 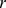 has the same effect as reducing the spacing and we find for 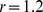 the spacing is 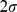. The figure of 3–4 fold overlap is quite commonly cited [30], as is the equivalent spacing of 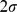 [40]. Thus our results match these overlap figures very closely.

Liu, Stevens and Sharpee [35] support our finding that perfect grid placement would give the best spatial resolution, but such precision is not found in biological cell arrays. They showed that the natural variation from the ideal grid location of field centres can be compensated by using irregular field shapes. Elliptical fields can have a more efficient packing density than circles and may be well suited for covering an array of irregular fields. By these means, irregular grids have been arranged to gain up to 92% of perfect placement.

For increasing overlap, several authors agree that a trade-off exists between better accuracy (and signal-to-noise performance) and redundancy, in that more connections are needed between maps, e.g. [39].

Regarding decoding from populations it is known that interpolation effects seem to be employed in the brain [41] and accurate spatial locations can be derived from the combination of firing grid cells [42]. Vector methods are widely advocated for this situation [42] but there is little significant data to guide the modeller on the performance and fidelity that can be achieved, especially when considering mapping transformations. There is some preference for linear vector summation methods [43] over vector averaging [22] but this is not yet resolved. Chaisanguanthum and Lisberger [36] support vector averaging as a simple, generic method that has been used successfully in various models. They performed a comparison of vector averaging with an inter-spike interval technique and with a maximum likelihood analysis; with all giving very similar results. They argue that a degree of sub-optimality in decoders is acceptable because neural systems already contain significant amounts of correlated noise that inevitably lowers precision. Hence, highly optimised performance will have diminishing returns in this area.

It is encouraging that these various relevant investigations strongly agree with our results, even though our approach is from a quite different, non-biological, perspective. This gives support to the existence of some general principles that will advance both robotic applications and neural modelling and understanding.
